# Supplementary material for: From inpatient to outpatient mental health care: Protocol for a randomised feasibility trial of a care transition intervention for patients with depression and anxiety (the AMBITION-trial)
Source: PLoS One. 2023 Nov 3;18(11):e0291067. doi: 10.1371/journal.pone.0291067 (PMC10624294; doi:10.1371/journal.pone.0291067)
Supplement: S3 File — (PDF) [file pone.0291067.s003.pdf]

Studienprotokoll

Übergang von stationärer in AMBulante psychosoziale Versorgung – Machbarkeit einer intersektoralen Care Transition IntervenTION bei Depression und Angst (AMBITION)

Studienleiter

Dr. med. Markus Haun, M. Sc. Psych., M.B.A.
Klinik für Allgemeine Innere Medizin und Psychosomatik
Universität Heidelberg
Thibautstrasse 4
D-69115 Heidelberg

Telefon +49 (0) 6221 - 56 - 38 39 6
Fax +49 (0) 6221 - 56 – 53 30
markus.haun@med.uni-heidelberg.de

Biometrikerin

Prof. Dr. sc. hum. Dipl.-Psych. Dipl.-Math. Beate Wild
Leitende Biometrikerin
Klinik für Allgemeine Innere Medizin und Psychosomatik
Universität Heidelberg
Im Neuenheimer Feld 410
D-69120 Heidelberg

Geldgeber

Zentralinstitut für die kassenärztliche Versorgung in der Bundesrepublik Deutschland, Stiftung des bürgerlichen Rechts, Salzufer 8, 10587 Berlin

Datum und Version des Protokolls

Datum: 05.05.2023, Version: 2.0

Unterschrift des Studienleiters

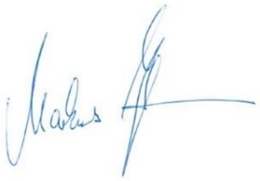

1. Zusammenfassung

Hintergrund

Patient*innen, die aufgrund depressiver Störungen oder Angststörungen stationär behandelt worden sind, finden aus vielerlei Gründen nach Entlassung aus stationärer Behandlung häufig nicht den Weg in die dringend indizierte leitliniengerechte ambulante Anschlussbehandlung. Dieser Bruch in der Kontinuität der Versorgung bedingt maßgeblich hohe Rehospitalisierungsraten bereits sehr kurz nach Entlassung aber auch im weiteren Verlauf mit. Mittel- bis langfristig birgt das für die Betroffenen das Risiko, dass sich die jeweilige Störung chronifiziert und die soziale Teilhabe (bspw. in der Familie oder am Arbeitsmarkt) nur noch vermindert oder im Extremfall gar nicht mehr möglich ist.

Ziel der Studie

Es soll eine Care Transition Intervention (CTI) auf Machbarkeit unter Studienbedingungen geprüft werden. Diese Intervention hat zum Ziel, Patient*innen einen flüssigeren Übergang zwischen dem stationären und ambulanten Sektor der psychosozialen Versorgung zu ermöglichen und damit Rehospitalisierungen zu vermeiden, mittelfristig soziale Teilhabe sicherzustellen und die Lebensqualität zu verbessern. Es wird explorativ im Zuge einer Machbarkeitsstudie geprüft, ob die Umsetzung der CTI möglich ist und dabei das Potential zeigt, die eben genannten Ziele zu verwirklichen, also letztlich zu einer Verbesserung der Versorgung beitragen kann.

Studiendesign und -durchführung

Durchgeführt wird eine individuell randomisiert-kontrollierte Machbarkeitsstudie. Dabei richtet sich die CTI an Patient*innen nach dem vollendeten 18. Lebensjahr mit klinisch bedeutsamer Depression und Angst am Übergang von stationärer in ambulante psychosoziale Versorgung. Insgesamt sollen 50 Proband*innen in die Studie eingeschlossen und im 1:1 Verhältnis in zwei Gruppen randomisiert werden. Zur Prä-Post-Evaluation werden sowohl Fragebogeninstrumente als auch qualitative Interviews eingesetzt. Die Auswertung erfolgt für die quantitativen Endpunkte deskriptiv, für die qualitativen Daten per softwaregestützter Inhaltsanalyse. Die Studie wird vor Einschluss der*des ersten Patient*in in einem Studienregister eingetragen.

2. Inhaltsverzeichnis

---

1. Zusammenfassung 2

2. Inhaltsverzeichnis 3

3. Einleitung/Wissenschaftliche Grundlagen 5

4. Ziele der Studie (allgemein) 6

5. Zielkriterien 7

5.1 Hauptzielkriterien 7

5.2 Nebenzielkriterien 7

6. Studienbedingte Maßnahmen 8

6.1 Rekrutierung und Aufklärung 8

6.2 Datenerhebungen 8

6.3 Behandlungsbedingungen 9

6.4 Studienablauf 13

6.5 Maßnahmen zur Qualitätssicherung 14

7. Erwarteter Nutzen 15

8. Mögliche Risiken oder Belastungen 15

9. Studientyp/Studiendesign 16

10. Ein- und Ausschlusskriterien 16

10.1 Einschlusskriterien 16

10.2 Ausschlusskriterien 16

11. Randomisierungsverfahren/-plan 17

12. Abbruchkriterien 17

12.1 Individuelle Abbruchkriterien 17

12.2 Abbruchkriterien für die Studie insgesamt 18

13. Statistisches Design 18

13.1 Statistische Methoden 18

13.2 Fallzahlplanung 18

14. Rechtliche und ethische Aspekte 19
14.1 Deklaration von Helsinki 19
14.2 Begutachtung durch die Ethikkommission 19
14.3 Angaben zur Freiwilligkeit der Teilnahme 19
14.4 Angaben zur Aufklärung und Einwilligung 19
14.5 Angaben zum Rücktrittsrecht und zur Datenvernichtung bei Rücktritt 19
14.6 Angaben zum Datenschutz 20
14.7 Angaben zu einer Genehmigung lt. StrlSchG 20
14.8 Finanzierung/institutionelle Verbindungen/Interessenkonflikte 20
14.9 Angaben zu Versicherungen (Wegeunfall/StrlSchV) 20
15. Literaturverzeichnis 21
16. Anhänge 24

3. Einleitung/Wissenschaftliche Grundlagen

Patient*innen, die aufgrund einer depressiven Störung und/oder Angststörung stationär behandelt worden sind, finden nach Entlassung aus stationärer Behandlung regelhaft nicht den Weg in die dringend indizierte leitliniengerechte ambulante Anschlussbehandlung.[1–4] Die Versäumung dieser ambulanten Behandlung führt zu hohen Raten von Rehospitalisierungen bereits sehr kurz nach Entlassung aber auch im weiteren Verlauf, reduzierter Medikamentenadhärenz, einem erhöhten Risiko der Chronifizierung der jeweiligen Störung und einer verminderten Teilhabe am Arbeitsmarkt.[4–7] Gründe für die ausbleibende Aufnahme der ambulanten Anschlussbehandlung finden sich auf mehreren Ebenen und reichen von strukturellen Barrieren wie einer geringen Verfügbarkeit von Behandlungsplätzen bis hin zu Barrieren auf der individuellen Ebene der Patient*innen wie ambivalenter Motivationslage gegenüber Weiterbehandlung, fehlendem Zugang zu Informationen über verfügbare Behandlungsangebote und daraus resultierenden Schwierigkeiten, sich im Gesundheitssystem zu orientieren.[8] Die Barrieren auf der individuellen Ebene der Patient*innen erscheinen als besonders hinderlich, gleichzeitig aber auch als vergleichsweise kurzfristig modifizierbar.[9] So zeigt sich, dass Patient*innen häufig Unterstützung benötigen, sich auf die Zeit nach dem stationären Aufenthalt vorzubereiten und im komplexen System der ambulanten psychosozialen Versorgung zurechtzufinden. Genau an diesem Punkt setzen sogenannte Care Transition Interventionen (CTI) an, die eine frühzeitig, respektive noch vor der Entlassung, einsetzende und für eine gewisse Zeit nach der Entlassung andauernde enge Begleitung dieser Patient*innen gewährleisten. Dabei wird zwischen unterschiedlichen Leistungserbringern vermittelt (integrierte Versorgung) und Patient*innen sollen befähigt werden, sich mit ihrer Störung weitergehend auseinanderzusetzen und selbstständig weitere Unterstützung in Anspruch zu nehmen (bspw. nach dem Ansatz der Patientenemanzipation durch Psychoedukation). Das Ziel von CTI ist, Patient*innen einen unterbrechungsfreien Übergang zwischen dem stationären und ambulanten Sektor der psychosozialen Versorgung zu ermöglichen und damit soziale Teilhabe (bspw. in der Familie oder am Arbeitsplatz) sicherzustellen und Rehospitalisierungen zu vermeiden.[10]

Um den Übergang von stationärer zu ambulanter psychosozialen Versorgung zu verbessern, wurden im deutschen Gesundheitssystem bisher Ansätze gewählt, die feste institutionelle Kooperationen zwischen Leistungserbringern fokussierten, die wiederum die Behandlungskontinuität fördern sollten.[11] Diese Ansätze bieten den Vorteil des Aufbaus von nachhaltigen Netzwerken von Behandler*innen in der entsprechenden Region, wodurch Patient*innen schneller überwiesen werden können, was wiederum zu Symptomverbesserungen, Lebensqualitätszuwachs und erhöhter Medikamentenadhärenz führt.[12] Als sehr problematisch zu sehen ist der hohe Aufwand im Vorfeld, den diese Netzwerke erfordern, und die verhältnismäßig geringe Übertragbarkeit auf andere sozialräumlich verschiedene Regionen, in denen möglicherweise

völlig andere Versorgungsressourcen vorherrschen. Die Schwierigkeit komplexerer Kooperation unterschiedlicher Beteiligter (z.B. Leistungserbringer, Krankenkassen, Verbände) zeigt sich daran, dass einige vielversprechende Projekte gestoppt und abgebrochen worden sind.[11] Aus diesem Grund erscheint der Einsatz von niederschwelligen CTI sinnvoll, in denen individuell mit den Patient*innen an der Schnittstelle zu den jeweiligen ambulanten Angeboten, die zur Verfügung stehen, gearbeitet wird. Ergebnisse aus Übersichtsarbeiten zu CTI zeigen, dass eine große Heterogenität besteht, was Ausgestaltung der Intervention, Zielgruppe und Outcomevariablen betrifft.[10,13,14] Beispielsweise umfasst das Spektrum der Interventionen Unterstützung beim Entlassungsprozess durch Peers und/oder Sozialarbeiter*innen bis hin zu einer kontinuierlichen Weiterbehandlung durch den*die Stationsärzt*in auch nach Beendigung des stationären Aufenthaltes. Nichtsdestotrotz haben sich CTI als effektiv und vor allem im Bereich der Psychosomatischen Medizin und Psychotherapie zusätzlich als kosteneffizient erwiesen.[15] Insbesondere reduzieren sie Rehospitalisierungen und verbessern sowohl die individuelle Lebensqualität der Patient*innen als auch den Wiedereinstieg in den Arbeitsmarkt im Vergleich zu Patient*innen, die in der Regelversorgung keine CTI erhalten.[10,16,17] Da diese vielversprechenden Ergebnisse hauptsächlich aus den USA stammen, wo die Grenzen zwischen dem stationären und ambulanten Sektor flexibler und weniger starr sind als im deutschen Gesundheitssystem, ist es angezeigt, CTI auch in Deutschland zu erproben, um so den Problemen bei der intersektoralen Versorgung zu begegnen.

4. Ziele der Studie (allgemein)

Ziel des beschriebenen Forschungsprojekts ist die Prüfung der Machbarkeit einer CTI zur niederschwelligen Unterstützung von Patient*innen mit depressiven Störungen und Angststörungen beim Übergang von stationärer zu ambulanter psychosozialer Versorgung. Dabei soll untersucht werden, ob sowohl Patient*innen als auch Leistungserbringer die Intervention akzeptieren und diese umgesetzt werden kann. Eine Nebenfragestellung zielt auf die Passung einzelner vorher festgelegter Interventionskomponenten ab. Ergebnisse dieses Projekts können dafür genutzt werden, die Intervention weiterzuentwickeln und hinsichtlich der Bedarfe der Patient*innen zu optimieren. Im Anschluss kann die Effektivität in einer ausreichend gepowerten randomisiert-kontrollierten Studie überprüft werden.

5. Zielkriterien

5.1 Hauptzielkriterien[18,19]

- Machbarkeit der Rekrutierung: Etablierung suffizienter Rekrutierungswege für Interventions- und Kontrollgruppe und Bestimmung der erforderlichen Ressourcen
  Operationalisierung über Recruitment Yield
- Machbarkeit der Randomisierung: Akzeptanz bei Patient*innen und Behandler*innen
  Operationalisierung über Consent Rate
- Angemessenheit (Appropriateness) und Machbarkeit der Intervention: Praktikabilität der Abläufe sowie Adhärenz von Proband*innen sowie des*der CTN
  Operationalisierung über Haltequote für beide Studienarme (Zahl der jeweils von den Proband*innen wahrgenommenen Interventionstermine), über quantitative und qualitative Erfassung von unerwünschten Ereignissen bzw. Effekten sowie über quantitative (regelmäßige systematische Abfrage zu einzelnen Interventionskomponenten) und qualitative Erfassung der Umsetzung (logistische und technische Realisierung der Intervention v.a. hinsichtlich Terminierung, Umsetzung der Intervention inkl. deren Dokumentation, Umsetzungstreue und Sicherheit der Patient*innen)
- Machbarkeit der Datenerhebung: Eignung der Instrumente zur Outcome-Messung (Responsiveness der Instrumente und Anteil der fehlenden Daten) und effektive Verblindung der Endpunkterheber*innen
  Operationalisierung über Exploration von Boden- und Deckeneffekte für die quantitativen Maße sowie über Bestimmung der Loss to Follow-up Raten und Zahl der ungeplanten vorzeitigen Entblindungen

5.2 Nebenzielkriterien

- Wiedereinweisungsquote (Rehospitalisierung)
- Inanspruchnahme psychosozialer Behandlungsangebote (Fragebogen zur Inanspruchnahme medizinischer und nicht medizinischer Versorgungsleistungen bei psychischen Erkrankungen, FIMPsy) [20]
- Psychische Symptomlast (Depressivität, Ängstlichkeit) (Patient Health Questionnaire Anxiety and Depression Scale, PHQ-ADS) [21]
- Lebensqualität (12-Item Short-Form Health Survey, SF-12) [22]
- Recovery (Recovery Assessment Scale, RAS-G) [23]

6. Studienbedingte Maßnahmen

6.1 Rekrutierung und Aufklärung

Die Rekrutierung von Patient*innen für AMBITION soll während ihres stationären Aufenthalts in der Klinik für Allgemeine Innere Medizin und Psychosomatik des Universitätsklinikums Heidelberg erfolgen. Geeignete Patient*innen werden (etwa) ab der Mitte ihres Behandlungszeitraums (entspricht regelhaft der fünften Behandlungswoche) durch ihre*n Bezugstherapeut*in im Rahmen der psychotherapeutischen Einzelgespräche ausgewählt, über die Studie informiert und mit den Studienunterlagen ausgestattet, bestehend aus Informationsschrift, Einwilligungserklärung in zweifacher Ausführung und dem Baseline-Fragebogenset. Stimmt die*der Patient*in schriftlich per Unterschrift zu, weitere Informationen erhalten zu wollen, wird sie*er innerhalb von zwei Tagen telefonisch durch die Studienzentrale kontaktiert. Die*der Patient*in wird (1) hinsichtlich der Erfüllung von Ein- und Ausschlusskriterien beurteilt, (2) bei Eignung zur Studienteilnahme ausführlich über die Studie aufgeklärt und hat (3) die ausführliche Möglichkeit, Fragen zu stellen. Schritt 1 und 2 werden nicht länger als 15 Minuten dauern. Ist kein Studieneinschluss möglich, wird dies mit Ablehnungsgrund, Alter und Geschlecht dokumentiert. Nach dem Aufklärungsgespräch durch die Studienzentrale füllen die an Studienteilnahme interessierten Patient*innen den Baseline-Fragebogen aus, unterschreiben die Einwilligungserklärung (Herstellung eines Informed Consent) und geben die Studienunterlagen im Teamstützpunkt der jeweiligen Station ab. Anschließend erfolgt innerhalb von 48 Stunden die Randomisierung und damit die Zuteilung der Proband*innen zu den beiden Studienarmen. Das Ergebnis der Zuteilung wird den Proband*innen telefonisch durch die*den CTN mitgeteilt. Bei Proband*innen, die der Interventionsbedingung (CTI) zugeordnet werden, erfolgt im Rahmen dieses Telefongesprächs bereits eine erste Terminvereinbarung mit der *dem CTN. Die*der CTN übermittelt das Ergebnis der Randomisierung an die*den jeweilige*n Bezugstherapeut*in.

6.2 Datenerhebungen

*Erhebungen bei den Proband*innen*

Die an der Studie teilnehmenden Patient*innen erhalten ein Fragebogenset, das aus folgenden validierten Fragebögen in deutscher Version besteht: Patient Health Questionnaire Anxiety and Depression Scale (PHQ-ADS),[21] 12-Item Short-Form Health Survey (SF-12)[22] und Recovery Assessment Scale (RAS-G).[23] Zusätzlich wird der Fragebogen zur Inanspruchnahme medizinischer und nicht medizinischer Versorgungsleistungen bei psychischen Erkrankungen (FIMPsy) eingesetzt, um Daten zur Nutzung von Gesundheitsleistungen jeglicher Art und Rehospitalisierungen zu gewinnen.[20] Die Patient*innen füllen diese Fragebögen erstmalig vor Randomisierung auf Station aus (wahlweise als auf dem

Postweg übermittelte Papierversion oder online über Unipark (Tivian XI GmbH)). Die Post-Erhebung erfolgt drei Monate und zwei weitere follow up-Erhebungen sechs bzw. neun Monate nach Einschluss in die Studie und werden von einem Mitglied des Studienteams durchgeführt, das hinsichtlich der Gruppenzugehörigkeit der Proband*innen verblindet ist (verblindete Endpunkterhebung). Um ein differenzierteres Bild hinsichtlich der poststationären Inanspruchnahme der Proband*innen zu gewinnen, werden bei den Post- und follow up-Erhebungen die Inanspruchnahme aller möglichen Angebote der ambulanten psychosozialen Versorgung abgefragt. Die Beantwortung der Fragebögen dauert jeweils ca. 15 bis 20 Minuten (Erfahrungswert aus ähnlichen vorangegangen Studien, bspw. PROVIDE-B).[24,25] Der spätmöglichste Termin für die Posterhebung bzw. follow up-Erhebungen beträgt 14 Tage nach dem eigentlich vorgesehenen Termin (drei, sechs oder neun Monate nach Einschluss). Nach der Post-Erhebung werden mit den Patient*innen der Interventionsbedingung qualitative Interviews zur Machbarkeit der Intervention durchgeführt (siehe Interviewleitfaden in Anhang A). Diese Interviews werden ca. 30 Minuten dauern und werden für die Auswertung aufgezeichnet und schnellstmöglich transkribiert. Die Audio-/Videoaufnahmen werden anschließend gelöscht.

*Erhebungen bei der*dem Care Transition Navigator (CTN)*

Nach Ende der Interventionsdurchführung wird ein Interview mit der*dem CTN durchgeführt. Dabei wird primär auf die Themen Angemessenheit, Machbarkeit und antizipierter Nutzen der Intervention fokussiert. Von besonderem Interesse sind dabei die Erfahrungen und Erkenntnisse der*des CTN während der Interventionsdurchführung (siehe Interviewleitfaden in Anhang B). Die Teilnahme an dem Interview erfordert den Informed Consent inkl. schriftlichem Einverständnis der*des CTN. Das Interview wird ca. 30 Minuten dauern und für die Auswertung aufgezeichnet und schnellstmöglich transkribiert. Die Audio-/Videoaufnahmen werden anschließend gelöscht.

6.3 Behandlungsbedingungen

Interventionsbedingung

Die Intervention, deren Machbarkeit geprüft werden soll, setzt sich aus Kernbestandteilen und optionalen Bestandteilen zusammen. Kernbestandteile sind Teile der Intervention, die obligat angewendet werden müssen. Die optionalen Bestandteile werden je nach Bedarf eingesetzt, wodurch die*der Proband*in individuell begleitet werden kann. Bei der Gestaltung der ambulanten psychosozialen Anschlusstherapie wird sich primär an der Empfehlung der behandelnden Stationsärzt*innen und Psychotherapeut*innen orientiert. Erster Kernbestandteil ist die Identifikation und das unmittelbar anschließende Adressieren von Barrieren, die die*den

Proband*in daran hindern, eine ambulante psychosoziale Anschlussbehandlung aufzunehmen. Dabei sollen die*der Proband*in durch motivierende Gesprächsführung und Psychoedukation dazu motiviert werden, nach dem Stationsaufenthalt Termine bei psychosozialen Anlaufstellen zu vereinbaren und wahrzunehmen. Die*der Proband*in wird dazu von einer*einem CTN betreut, wobei es sich dabei um eine organisatorische Unterstützung der Proband*innen und explizit nicht um eine psychotherapeutische Tätigkeit handelt. Als Vorbereitung auf die Interventionsdurchführung erhält die*der CTN im Vorfeld eine umfangreiche studienbedingte Schulung in motivierender Gesprächsführung und sozialarbeiterischen Themen (Kernbestandteil 2). Die*der CTN wird eine wissenschaftliche Hilfskraft mit einem Bachelorabschluss in Psychologie sein. Die*der Proband*in erarbeitet, angeleitet durch die*den CTN, eine subjektive Zusammenfassung des Stationsaufenthalts. Dadurch kann die*der CTN die Gewichtung von Herausforderungen aber auch von bisher Erreichtem aus Sicht der*des Proband*in erkennen und entsprechend zielgenauer in der weiteren gemeinsamen Arbeit adressieren (Kernbestandteil 3). Bspw. kann die*der ambulante Therapeut*in anhand von Faktoren ausgewählt werden, die beim*bei der jeweiligen Proband*in bereits während des stationären Aufenthaltes zu Verbesserung beigetragen haben. Diese Schritte erfolgen bereits vor der Entlassung der*des Proband*in.

Nach der Entlassung finden weitere Termine zwischen Proband*in und CTN statt, in denen kontinuierlich Barrieren auf Probandenseite identifiziert und adressiert werden und ggf. mit der*dem Proband*in die Ambivalenz gegenüber einer Anschlussbehandlung mit Ziel des Motivationsaufbaus reflektiert wird. Im ersten Monat nach Entlassung finden die Termine zwischen Proband*in und CTN mindestens alle zwei Wochen statt, in den Monaten zwei und drei nach Entlassung findet mindestens ein Termin pro Monat statt. Neben den Kernbestandteilen werden auch einige der optionalen Bestandteile Inhalt der Termine zwischen Proband*in und CTN sein. Bei probandenseitigem Bedarf nach einer stärkeren Strukturierung der Intervention wird ein patientenzentrierter schriftlicher Kurzentlassungsplan gemeinsam von Proband*in und CTN erarbeitet. Dieser sollte folgende Punkte beinhalten: (1) Klärung des Bedarfs und (2) der Ausgestaltung einer Anschlussbehandlung, (3) Identifizieren von möglichen Barrieren, diese Behandlung aufzunehmen, (4) Angebot, an welchen Stellen unterstützt werden kann, (5) Vereinbarung, wie häufig und in welchem Umfang Termine zwischen Proband*in und CTN stattfinden innerhalb der ersten drei Monate nach Entlassung (1. Monat mind. alle 2 Wochen; 2, und 3. Monat mind. ein Termin pro Monat). Zur Unterstützung und Motivation der*des Proband*in kann die*der CTN das Angebot an psychosozialen Behandler*innen in Wohnortnähe der*des Proband*in sichten. Des Weiteren sollte die*der Proband*in dazu motiviert werden, sich nach der Entlassung bei der*dem Hausärzt*in zu melden und über den weiteren Verlauf zu informieren. Weitere Bestandteile zur Unterstützung und Motivation können individuell

verschieden sein je nach Belastungsschwere und Ressourcen der*des Proband*in, werden aber Bestandteile des folgenden Spektrums beinhalten: systematische Erinnerungen an und Nachfragen zu Terminen und Medikamenteneinnahme, Screening auf Suizidalität, Evaluation von bekannten und/oder neu auftretenden Barrieren, Psychoedukation bezüglich des Umgangs mit psychischer Störung und Bewältigungsstrategien und Verhaltensaktivierung (positive Einstellung bewahren, Lösungsorientierung, Anregung zur Selbsthilfe). Diese optionalen Bestandteile haben sich in mehreren vergleichbaren Studien als wirksam erwiesen.[10,26] Wenn ein persönliches Treffen nicht möglich ist, werden die Termine zwischen Proband*in und CTN per Videokonsultation durchgeführt. Es ist geplant, dass die Termine zwischen Proband*in und CTN zwischen 30 und 60 Minuten dauern.

Begleitend wird die*der CTN alle zwei Wochen eine oberärztlich geleitete Fallsupervision in Anspruch nehmen, bei der alle Proband*innen besprochen werden. Insbesondere wird thematisiert werden, wie mit schwer erreichbaren und wenig adhärenten Proband*innen umgegangen werden könnte, um auch in dieser Gruppe den Übergang in ambulante psychosoziale Anschlussbehandlung erfolgreich umzusetzen (Kernbestandteil 4).

Tabellarische Interventionsbeschreibung

Kernbestandteile	Wirken/Nutzen
**Management von Barrieren**	Identifizieren/Adressieren von Barrieren bei der Vorbereitung einer Anschlussbehandlung
**Betreuung durch geschulten Care Transition Navigator**	Expertise in häufig auftretenden Problemfeldern, Unterstützung bei der Orientierung im Behandlungssystem
**Reflexion des Stationsaufenthaltes**	Fokussieren auf als wichtig wahrgenommene Aspekte aus Proband*innensicht
**Supervision**	Qualitätssicherung der Betreuung bei schwer kooperierenden Proband*innen
**Optionale Bestandteile**	**Wirken/Nutzen**
**Schriftl. Kurzentlassungsplan**	Gemeinsame Erarbeitung von zu fokussierenden Aspekten
**Systematisches Erinnern**	Unterstützung zur Behandlungskontinuität
**Screening auf Suizidalität,**	rechtzeitiges Erkennen von selbstgefährdendem Verhalten
**Psychoedukation bezüglich des Umgangs mit psychischer Störung**	Nachhaltiger selbstständiger Umgang mit möglicherweise chronischen Belastungen
**Verhaltensaktivierung**	positive Einstellung bewahren, Lösungsorientierung, Anregung zur Selbsthilfe

Kontrollbedingung

Proband*innen der Kontrollbedingung werden nach Maßgabe des*der Bezugstherapeut*in bei der Suche nach einer ambulanten psychosozialen Anschlussbehandlung unterstützt (z.B. durch Aushändigung von Kontaktdaten von ambulanten Psychotherapeut*innen in der Umgebung und Motivation zur Kontaktaufnahme im weiteren stationären Behandlungsverlauf). Dies kann auch – muss aber nicht – die Aufnahme in die Nachbetreuungsgruppe der Station bedeuten. Im Rahmen dieses Angebots wird sich über einen Zeitraum von fünf Wochen nach Entlassung einmal wöchentlich in einem Gruppenformat getroffen, um Probleme bei der Organisation der Anschlussbehandlung zu erörtern und allgemeine und wenig individualisierte Unterstützung zu gewährleisten.

6.4 Studienablauf

Schematischer Zeitplan (nach SPIRIT 2013 Statement)

			**STUDIENABSCHNITT**							
	**Einschluss**	**Zuteilung**	**Post-Zuteilung**					**follow-up**		**Close-out**
**ZEITPUNKT**	t_0	t_1	t_{1a}	t_{1b}	t_{1c}	t_{1d}	t_2 **(t_1+3 Monate)**	t_3 **(t_1+6 Monate)**	t_4 **(t_1+9 Monate)**	t_x
EINSCHLUSS:										
Screening nach Ein- und Ausschlusskriterien	X									
Informed Consent	X									
Baseline-Erhebung	X									
Randomisierung		X								
Zuteilung		X								
INTERVENTIONEN:										
Care Transition Intervention (CTI)			←	—	—	→				
Treatment as usual			←	—	—	→				
ERHEBUNGEN:										
Soziodemografie	X									
PHQ-ADS, SF-12, RAS-G, FIMPsy	X						X	X	X	
*Interviews: CTN und Patient*innen aus der Interventionsbedingung*							X			

Für eine Studienteilnahme in Frage kommende Patient*innen werden während ihres stationären Aufenthalts im Rahmen der Einzelpsychotherapie auf die Studie angesprochen und mit den Studienunterlagen ausgestattet. Im Falle einer schriftlichen Einwilligung zur Kontaktaufnahme werden die Patient*innen innerhalb von zwei Tagen durch das Studienteam angesprochen, um hinsichtlich der Ein- und Ausschlusskriterien beurteilt und ausführlich über die Studie aufgeklärt zu werden (t_0 im schematischen Zeitplan). Sobald der unterschriebene Informed Consent sowie der ausgefüllte Baseline-Fragebogen dem Studienteam vorliegen, erfolgt innerhalb von weiteren zwei Tagen die Randomisierung und Zuteilung der Patient*innen (t_1). Der anschließende Interventionszeitraum bemisst sich auf drei Monate (t_{1a-d} repräsentativ für vier Termine der CTI). Proband*innen der Interventionsbedingung erhalten die Care Transition Intervention durch die*den CTN. Proband*innen der Kontrollbedingung werden im stationären Behandlungsverlauf durch die*den Bezugstherapeut*in und ggf. poststationär im Rahmen der Nachbetreuungsgruppe bei der Suche nach einer ambulanten psychosozialen Anschlussbehandlung unterstützt. Die Post-Erhebung erfolgt drei Monate nach Studieneinschluss (t_2). Zwei quantitative Follow-up-Erhebungen sind sechs (t_3) und neun Monate (t_4) nach Einschluss geplant. Qualitative Interviews mit der*dem CTN und den Proband*innen der Interventionsbedingung werden nach Ende der Interventionszeitraum (CTN) bzw. im Anschluss an die Posterhebung (t_2) geführt (Proband*innen).

6.5 Maßnahmen zur Qualitätssicherung

- Adhärenzprüfung: Nach jedem Interventionstermin wird die*der CTN einen Online-Fragebogen ausfüllen, in dem abgefragt wird, welche Interventionskomponenten in der jeweiligen Sitzung angewendet wurden. Darüber hinaus ist die*der CTN angehalten, während der gesamten Intervention eine detaillierte Interventionsdokumentation zu führen.
- Supervision: Die*der CTN wird während der Studie durch eine*einen Oberärzt*in der Klinik für Allgemeine Innere Medizin und Psychosomatik supervidiert. Die Supervision findet in 14-tägigem Rhythmus statt.
- Datenmanagement: Um Dateneingabefehler zu minimieren, werden die Eingabefelder so programmiert, dass lediglich gültige Werte eingegeben werden können. Eingegebene Datensätze werden kontinuierlich auf Konsistenz mit bereits eingegebenen Daten überprüft. Die Datenbereinigung wird von zwei Mitgliedern des Studienteams unabhängig voneinander durchgeführt.

7. Erwarteter Nutzen

Eine CTI kann dazu beitragen, die gesundheitsbezogene Lebensqualität von Patient*innen zu erhöhen und gleichzeitig Rehospitalisierungen von kürzlich entlassenen psychisch erkrankten Patient*innen zu reduzieren[10]. Konkret bedeutet das, dass Proband*innen in der Interventionsbedingung durch die zusätzliche Unterstützung bei der Organisation der ambulanten psychosozialen Anschlussbehandlung weniger häufig wieder in stationäre Behandlung aufgenommen werden müssen und sich durch die kontinuierliche Behandlung eine Symptomverbesserung ergibt. Proband*innen in der Kontrollbedingung erhalten kein zusätzliches Angebot an Unterstützung, es werden allerdings auch keine Maßnahmen außerhalb der Studie ausgeschlossen. Auf einer gesellschaftlichen Ebene kommen Analysen zur Kosteneffizienz vergleichbarer Interventionen zu unterschiedlichen Ergebnissen. In einer Studie werden die Kosten für ein Zugewinn an Lebensqualität als zu hoch angesehen.[28] Eine andere intensivere CTI hingegen zeigte nicht nur einen positiven klinischen Effekt, sondern war auch kostengünstiger als die Regelversorgung.[15] Im vorgelegten Projekt soll die Machbarkeit einer CTI untersucht werden, um im Falle gegebener Machbarkeit anschließend die Wirksamkeit und Effizienz in einer größer angelegten randomisiert-kontrollierten Interventionsstudie mit gesundheitsökonomischer Evaluation (Piggy-Back Design) zu testen. Um im Hinblick auf diese potentielle große konfirmatorische Studie unnötige Kosten zu vermeiden, die Intervention möglichst effizient zu gestalten und bestmöglich nach den Bedürfnissen der Patient*innen auszurichten, ist es wichtig, die Machbarkeit und Akzeptanz der Intervention im Vornhinein im Zuge einer Machbarkeitsstudie zu testen.

8. Mögliche Risiken oder Belastungen

Es ist nicht davon auszugehen, dass der Einsatz der beschriebenen CTI mit Risiken und/oder Nachteilen für die Proband*innen einhergeht, da die Intervention einen Zusatz an Unterstützung bei der Suche nach einer ambulanten psychosozialen Anschlussbehandlung vorsieht und dadurch keine Bestandteile der routinemäßigen Versorgung beschnitten werden. Lediglich die im Rahmen der Intervention allenfalls sporadisch vorgesehene Beschäftigung mit möglicherweise problematischen Lebensinhalten kann manchmal mit vorübergehendem Selbstzweifel, zwischenzeitlicher Symptomverschlechterung und Veränderungen in persönlichen Beziehungen einhergehen. Dies sind natürliche Reaktionen und kein Zeichen eines ungünstigen Verlaufs. Vor dem Hintergrund der verhältnismäßig geringen Intensität der Intervention sind diese Risiken aber eher unwahrscheinlich. Das Ausfüllen der Instrumente im Rahmen der Erhebung der Haupt- und Nebenzielkriterien wird für Baseline- und Posterhebung jeweils 20 Minuten nicht überschreiten.

9. Studientyp/Studiendesign

Es handelt sich um eine monozentrische, prospektive, Endpunkterheber-verblindete und individuell randomisiert-kontrollierte Machbarkeitsstudie mit qualitativer Prozessevaluation (Interviews mit Proband*innen sowie dem CTN). Dabei werden in der Klinik für Allgemeine Innere Medizin und Psychosomatik am Universitätsklinikum Heidelberg stationär behandelte Patient*innen mit Depression und/oder Angststörung beim Übergang in den ambulanten Sektor der psychosozialen Versorgung durch einen CTN unterstützt. Der Interventionszeitraum beträgt drei Monate nach Entlassung von der psychotherapeutischen Station. Datenerhebungen erfolgen bei Einschluss in die Studie vor Randomisierung (Baseline-Erhebung) sowie drei (Posterhebung), sechs und neun Monate (follow up-Erhebungen) nach Entlassung. Da die Prüfung der Machbarkeit im Vordergrund steht, handelt es sich um eine deskriptive Studie ohne Hypothesentestung.

10. Ein- und Ausschlusskriterien

10.1 Einschlusskriterien für Patient*innen

- Empfehlung der behandelnden Stationsärzt*innen und/oder Psychotherapeut*innen, eine ambulante psychosoziale Anschlussbehandlung aufzunehmen
- vollendetes 18. Lebensjahr
- stationäre Behandlung aufgrund einer mindestens mittelgradigen Depression und/oder Angststörung am Universitätsklinikum Heidelberg
- schriftlich dokumentierter Informed Consent/Einverständniserklärung
- Einwilligungsfähigkeit

10.2 Ausschlusskriterien für Patient*innen

- geplante Wiederaufnahme einer bereits vor dem aktuellen stationären Aufenthalt angelaufenen ambulanten Richtlinienpsychotherapie (Kurzzeit- oder Langzeittherapie) nach Stationsaufenthalt
- aktive Suizidgedanken oder aktive Gedanken, anderen Gewalt anzutun
- akute psychotische Zustandsbilder
- Demenz und/oder schwere kognitive Beeinträchtigung
- unzureichende deutsche Sprachkenntnisse

10.3 Einschlusskriterien für die*den CTN

- berufsqualifizierender Abschluss in einem der folgenden Berufsfelder: Medizin, Psychologie, Pflege, Gesellschafts-, Geistes- und Sprachwissenschaften, Sozialwesen
- hervorragende deutsche Sprachkenntnisse
- schriftlich dokumentierter Informed Consent/Einverständniserklärung

10.4 Ausschlusskriterien für die*den CTN

- keine Volljährigkeit

11. Randomisierungsverfahren/-plan

Die Patient*innen werden zufällig einer der beiden Studienbedingungen (Unterstützung durch CTN vs. Treatment-as-Usual, TAU, d.h. gewöhnliches Entlassungsmanagement ohne spezielle zusätzliche Unterstützung bei der Suche nach einer Anschlusstherapie) im Verhältnis 1:1 zugeteilt. Die Randomisierung erfolgt nach Einholung des Informed Consent und nicht später als 28 Tage nach der letzten Datenerhebung (Screening oder Baseline) unabhängig durch die Studienzentrale (Stephanie Estel, Klinik für Allgemeine Innere Medizin und Psychosomatik, Universität Heidelberg). Es wird das webbasierte Programm *Randomizer Version 2.1.0* des Instituts für Medizinische Informatik, Statistik und Dokumentation der Medizinischen Universität Graz (https://www.randomizer.at) verwendet, welches durch zentrale Randomisierung die Geheimhaltung der Behandlungsfolge bis zur Zuteilung gewährleistet. Die Erzeugung der Behandlungsfolge erfolgt über eine computergenerierte Sequenz von Zufallszahlen. Es kommt Blockrandomisierung mit Stratifizierung der Patient*innen nach Baseline-Wert im PHQ-ADS (Depressivität und Angst) zur Anwendung.

12. Abbruchkriterien

12.1 Individuelle Abbruchkriterien

Als Abbruchkriterium wird der Widerruf der Zustimmung der*des Proband*in zur Studienteilnahme definiert, wobei die Einwilligung zur Studienteilnahme zu jedem Zeitpunkt und ohne Angabe von Gründen zurückgezogen werden kann. Bei Rücktritt von der Studie werden die Proband*innen gefragt, ob sie mit der Auswertung der Daten einverstanden sind. Falls sie damit nicht einverstanden sind, werden die Daten vernichtet.

12.2 Abbruchkriterien für die Studie insgesamt

Keine.

13. Statistisches Design

13.1 Statistische Methoden

Es handelt sich um eine deskriptive Studie, deren primäres Ziel die Erprobung und Feststellung der Machbarkeit einer CTI ist. Die Machbarkeit und Akzeptanz der Interventions- und Studienabläufe werden anhand der oben dargestellten Kriterien beurteilt. Der Baseline-Post-Vergleich der oben angeführten sekundären Auswertungsinhalte bei den Proband*innen erfolgt primär deskriptiv unter Bestimmung von zentralen Lagemaßen (Mittelwert, Median) und Darstellung von Variabilitätsmaßen (Standardabweichung, Interquartilsabstand und Range) sowie absoluter und relativer Häufigkeiten. Im Anschluss an eine deskriptive Aufbereitung sollen Unterschiedstendenzen in den o. g. Auswertungsinhalten (abhängige Variablen) zwischen Interventions- und Kontrollbedingung (unabhängige Variable) bestimmt werden. Hierzu werden Mittelwertsdifferenzen sowie Effektstärken (nach Cohen) zusammen mit den jeweiligen 95 %-Konfidenzintervallen berechnet. Da die Studie rein explorativen Charakter hat, besitzt die Analyse keinen konfirmatorischen Wert. Die Auswertung wird auch eine nähere Betrachtung der Non-Responder und Ablehner*innen (inkl. der Ablehnungsgründe) im Vergleich zu den eingeschlossenen Proband*innen umfassen. Es werden hierbei zum Gruppenvergleich analoge, statistische Verfahren eingesetzt. Sollten Proband*innen die Studie abbrechen, erfolgt eine separate Beschreibung dieser Stichprobe, um Hinweise auf eine eventuelle Selektivität zu gewinnen. Die gesamte statistische Auswertung wird in R (Version 4.1.0 oder höher) erfolgen. Die Studie wird vor Einschluss der*des ersten Proband*in in ein Studienregister (WHO International Clinical Trials Registry) eingetragen.

13.2 Fallzahlplanung

Die Stichprobengröße beträgt 50 Proband*innen, die im 1:1 Verhältnis entweder der Interventionsbedingung, die die CTI enthält, oder der Kontrollbedingung, die die übliche Versorgung im Anschluss an einen stationären Aufenthalt enthält, zufällig zugeteilt werden. Da es sich bei der beantragten Studie um eine Machbarkeitsstudie handelt, die rein explorativen Charakter hat und keine konfirmatorischen Analysen vorsieht, erfolgte keine endpunktgetriebene Fallzahlplanung. Es wurde sich an Empfehlungen orientiert, die für Studien, die primär Studienprozesse auf Machbarkeit testen, eine Stichprobengröße von 50 Proband*innen

vorschlagen.[31] Unter Berücksichtigung der Anzahl entlassener Patient*innen von den entsprechenden Stationen wird mit einem Rekrutierungszeitraum von drei Monaten gerechnet.

14. Rechtliche und ethische Aspekte

14.1 Deklaration von Helsinki

Die Untersuchung wird in Übereinstimmung mit der Deklaration von Helsinki und der Berufsordnung für Ärzt*innen der Landesärztekammer Baden-Württemberg in den jeweils aktuellen Fassungen durchgeführt. Die ICH-GCP-Guidelines werden berücksichtigt.

14.2 Begutachtung durch die Ethikkommission

Das Studienprotokoll wird vor Studienbeginn der Ethikkommission der Medizinischen Fakultät Heidelberg zur Begutachtung vorgelegt. Es wird nicht mit dem Einschluss von Proband*innen begonnen, bevor nicht das schriftliche, zustimmende Votum der Ethikkommission vorliegt.

14.3 Angaben zur Freiwilligkeit der Teilnahme

Die Teilnahme der Proband*innen ist freiwillig.

14.4 Angaben zur Aufklärung und Einwilligung

Die Studienteilnehmer*innen werden vor Studienbeginn schriftlich und mündlich über Wesen und Tragweite der geplanten Untersuchung, insbesondere über den möglichen Nutzen für ihre Gesundheit und eventuelle Risiken, aufgeklärt. Ihre Zustimmung wird durch Unterschrift auf der Einwilligungserklärung dokumentiert.

14.5 Angaben zum Rücktrittsrecht und zur Datenvernichtung bei Rücktritt

Die Zustimmung kann von der*dem Proband*in jederzeit, ohne Angaben von Gründen und ohne Nachteile für die weitere medizinische Versorgung zurückgezogen werden.
Bei Rücktritt von der Studie wird die*der Proband*in gefragt, ob sie*er mit der Auswertung der Daten einverstanden ist. Falls sie*er damit nicht verstanden ist, werden die Daten vernichtet. Sollte keine Klärung möglich sein, wird bereits gewonnenes Datenmaterial vernichtet.

14.6 Angaben zum Datenschutz

Die Namen der Proband*innen sowie alle anderen vertraulichen Informationen unterliegen der ärztlichen Schweigepflicht und den Bestimmungen der Datenschutz-Grundverordnung (DSGVO) sowie des Landes- bzw. Bundesdatenschutzgesetzes (LDSG bzw. BDSG). Eine Weitergabe von Daten der Proband*innen erfolgt ggf. nur in pseudonymisierter Form. Die Pseudonymisierung der Daten erfolgt bereits vor Ausgabe der Fragebögen. Der Pseudonymisierungsschlüssel wird durch den Studienleiter vertraulich verwaltet und verbleibt bei diesem. Dritte erhalten keinen Einblick in Originalunterlagen. Bei Veröffentlichungen, z. B. in Fachzeitschriften, kann und wird kein Bezug zu einzelnen Personen hergestellt werden. Die in der Studie erhobenen bzw. gewonnenen Daten werden nach den geltenden Richtlinien bis zehn Jahre nach Erhebungsdatum aufbewahrt und anschließend vernichtet. Sobald wie möglich, jedoch spätestens nach Abschluss aller Publikationen erfolgt gem. § 35 Abs. 2 LDSG BW die Anonymisierung der personenbezogenen Daten.

14.7 Angaben zu einer Genehmigung lt. StrlSchG

Nicht zutreffend.

14.8 Finanzierung/institutionelle Verbindungen/Interessenkonflikte

Die Studie wird vom Zentralinstitut für die kassenärztliche Versorgung in der Bundesrepublik Deutschland, Stiftung des bürgerlichen Rechts, gefördert. Es bestehen keine Interessenskonflikte zwischen den Mitgliedern des Studienteams und Dritten.

14.9 Angaben zu Versicherungen (Wegeunfall/StrlSchV)

Nicht erforderlich.

15. Literaturverzeichnis

1. Jørgensen K, Bonde Dahl M, Frederiksen J. Healthcare Professionals' and Users' Experiences of Intersectoral Care between Hospital and Community Mental Healthcare. *Int J Environ Res Public Health*. 2020;17(18). doi:10.3390/ijerph17186510

2. Ko Y, Park S. Life after hospital discharge for people with long-term mental disorders in South Korea: Focusing on the "revolving door phenomenon." *Perspectives in Psychiatric Care*. 2021;57(2):531-538. doi:https://doi.org/10.1111/ppc.12575

3. Neiterman E, Wodchis WP, Bourgeault IL. Experiences of Older Adults in Transition from Hospital to Community*. *Canadian Journal on Aging / La Revue canadienne du vieillissement*. 2015;34(1):90-99. doi:10.1017/S0714980814000518

4. Wiegand HF, Saam J, Marschall U, et al. Challenges in the Transition from In-Patient to Out-Patient Treatment in Depression. *Deutsches Aerzteblatt Online*. Published online July 6, 2020. doi:10.3238/arztebl.2020.0472

5. Niimura J, Tanoue M, Nakanishi M. Challenges following discharge from acute psychiatric inpatient care in Japan: patients' perspectives. *Journal of Psychiatric and Mental Health Nursing*. 2016;23(9-10):576-584. doi:https://doi.org/10.1111/jpm.12341

6. Olfson M, Marcus SC, Doshi JA. Continuity of Care After Inpatient Discharge of Patients With Schizophrenia in the Medicaid Program: A Retrospective Longitudinal Cohort Analysis. *J Clin Psychiatry*. 2010;71(7):0-0. doi:10.4088/JCP.10m05969yel

7. Wright N, Rowley E, Chopra A, Gregoriou K, Waring J. From admission to discharge in mental health services: a qualitative analysis of service user involvement. *Health Expectations*. 2016;19(2):367-376. doi:https://doi.org/10.1111/hex.12361

8. Storm M, Husebø AML, Thomas EC, Elwyn G, Zisman-Ilani Y. Coordinating Mental Health Services for People with Serious Mental Illness: A Scoping Review of Transitions from Psychiatric Hospital to Community. *Adm Policy Ment Health*. 2019;46(3):352-367. doi:10.1007/s10488-018-00918-7

9. Nolan P, Bradley E, Brimblecombe N. Disengaging from acute inpatient psychiatric care: a description of service users' experiences and views. *Journal of Psychiatric and Mental Health Nursing*. 2011;18(4):359-367. doi:10.1111/j.1365-2850.2010.01675.x

10. Hegedüs A, Kozel B, Richter D, Behrens J. Effectiveness of Transitional Interventions in Improving Patient Outcomes and Service Use After Discharge From Psychiatric Inpatient Care: A Systematic Review and Meta-Analysis. *Front Psychiatry*. 2020;10. doi:10.3389/fpsyt.2019.00969

11. Schmid P, Steinert T, Borbé R. Systematische Literaturübersicht zur Implementierung der sektorübergreifenden Versorgung (Regionalbudget, integrierte Versorgung) in Deutschland. *Psychiatr Prax*. 2013;40(8):414-424. doi:10.1055/s-0033-1343192

12. Lambert M, Bock T, Schöttle D, et al. Assertive Community Treatment as Part of Integrated Care Versus Standard Care: A 12-Month Trial in Patients With First- and Multiple-Episode Schizophrenia Spectrum Disorders Treated With Quetiapine Immediate Release (ACCESS Trial). *J Clin Psychiatry*. 2010;71(10):0-0. doi:10.4088/JCP.09m05113yel

13. Gaebel W, Kerst A, Janssen B, et al. EPA guidance on the quality of mental health services: A systematic meta-review and update of recommendations focusing on care coordination. *Eur Psychiatry*. 2020;63(1). doi:10.1192/j.eurpsy.2020.75

14. Vigod SN, Kurdyak PA, Dennis CL, et al. Transitional interventions to reduce early psychiatric readmissions in adults: systematic review. *The British Journal of Psychiatry*. 2013;202(3):187-194. doi:10.1192/bjp.bp.112.115030

15. Moessner M, Bauer S, Özer F, Wolf M, Zimmer B, Kordy H. Cost-effectiveness of an Internet-based aftercare intervention after inpatient treatment in a psychosomatic hospital. *Psychotherapy Research*. 2014;24(4):496-503. doi:10.1080/10503307.2013.845919

16. Jackson C, DuBard A, Swartz M, et al. Readmission Patterns and Effectiveness of Transitional Care Among Medicaid Patients With Schizophrenia and Medical Comorbidity. *North Carolina Medical Journal*. 2015;76(4):219-226. doi:10.18043/ncm.76.4.219

17. Reynolds W, Lauder W, Sharkey S, Maciver S, Veitch T, Cameron D. The effects of a transitional discharge model for psychiatric patients. *Journal of Psychiatric and Mental Health Nursing*. 2004;11(1):82-88. doi:https://doi.org/10.1111/j.1365-2850.2004.00692.x

18. Proctor E, Silmere H, Raghavan R, et al. Outcomes for Implementation Research: Conceptual Distinctions, Measurement Challenges, and Research Agenda. *Adm Policy Ment Health*. 2011;38(2):65-76. doi:10.1007/s10488-010-0319-7

19. Eldridge SM, Lancaster GA, Campbell MJ, et al. Defining Feasibility and Pilot Studies in Preparation for Randomised Controlled Trials: Development of a Conceptual Framework. *PLOS ONE*. 2016;11(3):e0150205. doi:10.1371/journal.pone.0150205

20. Grupp H, König HH, Riedel-Heller S, Konnopka A. FIMPsy – Fragebogen zur Inanspruchnahme medizinischer und nicht medizinischer Versorgungsleistungen bei psychischen Erkrankungen: Entwicklung und Verwendung. *Psychiatr Prax*. 2018;45(02):87-94. doi:10.1055/s-0042-118033

21. Kroenke K, Spitzer RL, Williams JBW, Löwe B. The Patient Health Questionnaire Somatic, Anxiety, and Depressive Symptom Scales: a systematic review. *General Hospital Psychiatry*. 2010;32(4):345-359. doi:10.1016/j.genhosppsych.2010.03.006

22. Ware JE, Kosinski M, Keller SD. A 12-Item Short-Form Health Survey: Construction of Scales and Preliminary Tests of Reliability and Validity. *Medical Care*. 1996;34(3):220-233.

23. Cavelti M, Wirtz M, Corrigan P, Vauth R. Recovery assessment scale: Examining the factor structure of the German version (RAS-G) in people with schizophrenia spectrum disorders. *Eur Psychiatry*. 2017;41:60-67. doi:10.1016/j.eurpsy.2016.10.006

24. Tönnies J, Hartmann M, Wensing M, et al. Mental health specialist video consultations for patients with depression or anxiety disorders in primary care: protocol for a randomised controlled feasibility trial. *BMJ Open*. 2019;9(9). doi:10.1136/bmjopen-2019-030003

25. Tönnies J, Hartmann M, Wensing M, et al. Mental Health Specialist Video Consultations Versus Treatment-as-Usual for Patients With Depression or Anxiety Disorders in Primary Care: Randomized Controlled Feasibility Trial. *JMIR Mental Health*. 2021;8(3):e22569. doi:10.2196/22569

26. Steffen S, Kösters M, Becker T, Puschner B. Discharge planning in mental health care: a systematic review of the recent literature. *Acta Psychiatrica Scandinavica*. 2009;120(1):1-9. doi:10.1111/j.1600-0447.2009.01373.x

27. Abu HO, Anatchkova MD, Erskine NA, et al. Are we “missing the big picture” in Transitions of Care? Perspectives of Healthcare Providers Managing Patients with Unplanned Hospitalization. *Appl Nurs Res*. 2018;44:60-66. doi:10.1016/j.apnr.2018.09.006

28. Puschner B, Baumgartner I, Loos S, et al. Kosteneffektivität bedarfsorientierter Entlassungsplanung bei Menschen mit hoher Inanspruchnahme psychiatrischer Versorgung. *Psychiatr Prax*. 2012;39(08):381-387. doi:10.1055/s-0032-1327188

29. Jacobi F, Höfler M, Siegert J, et al. Twelve-month prevalence, comorbidity and correlates of mental disorders in Germany: the Mental Health Module of the German Health Interview and Examination Survey for Adults (DEGS1-MH). *Int J Methods Psychiatr Res*. 2014;23(3):304-319. doi:10.1002/mpr.1439

30. Wittchen HU, Jacobi F. *Angststörungen*. Nachdr. Robert Koch-Inst; 2007.

31. National Insitute for Health Research. Justify sample size for a feasibility study. RDS London. Accessed June 28, 2021. https://www.rds-london.nihr.ac.uk/resources/justify-sample-size-for-a-feasibility-study/

16. Anhänge

Anhang A. Interviewleitfaden für Interviews mit Patient*innen zu t_2

A. Einführung und Projektvorstellung

- Vorstellen Interviewer*in und Protokollant*in
- Hinweis auf Audio- und Videoaufzeichnung, Datenschutz, Einholung schriftlicher Informed Consent
- Schilderung des Interviewablaufs
- Einholung demographischer Informationen: Alter, Schulabschluss, Partnerschaft

B. Inhaltliche Fragen

- Wie zufrieden sind Sie aktuell mit Ihrer gesundheitlichen Situation?
- Wie zufrieden sind Sie mit der Unterstützung durch die/den Care Transition Navigator?
- Wie haben Sie die Kommunikation zwischen Ihnen und der/dem Care Transition Navigator?
- Wie patientenfreundlich haben Sie die Unterstützung nach diesem Ansatz erlebt?
- Würden Sie anderen diese Art der Unterstützung empfehlen?
- Wie empfanden Sie die Beziehung mit der der/dem Care Transition Navigator?
- Wie beurteilen Sie den Zugang zu psychosozialer Unterstützung im Lichte der Care Transition Intervention?
- Was an der Unterstützung durch die/den Care Transition Navigator war für Sie besonders hilfreich?
- Gibt es etwas, dass Sie in der Umsetzung anders machen würden?

C. Abschluss

- Gibt es Aspekte, die Ihnen noch wichtig sind und wir noch nicht thematisiert haben? Haben Sie noch Fragen?

Anhang B. Interviewleitfaden für Interviews mit der/dem Care Transition Navigator zu t_2

A. Einführung und Projektvorstellung

- Vorstellen Interviewer*in und Protokollant*in
- Hinweis auf Audio- und Videoaufzeichnung, Datenschutz, Einholung schriftlicher Informed Consent
- Schilderung des Interviewablaufs
- Einholung demographischer Informationen: Alter, Schulabschluss, Partnerschaft

B. Inhaltliche Fragen

- Wenn Sie an Ihre positiven und negativen Erfahrungen mit der Care Transition Intervention denken, wie sieht Ihre Bilanz aus?
- Wie effektiv haben Sie die Unterstützung für die Patientinnen und Patienten erlebt?
- Welche Rückmeldungen haben Sie von den Patientinnen und Patienten erhalten?
- Wie haben Sie den Übergang aus der stationären Behandlung der Patientinnen und Patienten erlebt?
- Wie praktikabel war die Terminfindung?
- Wie beurteilen Sie die Begegnung mit Patientinnen per Videokonsultation? (wenn stattgefunden)
- Wie zufrieden sind Sie mit dem Management von Notfällen?
- Wie zufrieden waren Sie mit dem Umfang, in dem Sie dem Studienteam Feedback geben konnten?
- Inwiefern haben Sie Verbesserungen in der Motivation und Eigenständigkeit der Patientinnen und Patienten wahrgenommen?

C. Abschluss

- Gibt es Aspekte, die Ihnen noch wichtig sind und wir noch nicht thematisiert haben?
- Haben Sie noch Fragen?
