# Supplementary material for: From inpatient to outpatient mental health care: Protocol for a randomised feasibility trial of a care transition intervention for patients with depression and anxiety (the AMBITION-trial)
Source: PLoS One. 2023 Nov 3;18(11):e0291067. doi: 10.1371/journal.pone.0291067 (PMC10624294; doi:10.1371/journal.pone.0291067)
Supplement: S4 File — (PDF) [file pone.0291067.s004.pdf]

Studyprotocol

From inpatient to outpatient mental health care: Protocol for a randomised feasibility trial of a care transition intervention for patients with depression and anxiety (the AMBITION-trial)

Director of Study

Dr. med. Markus Haun, M. Sc. Psych., M.B.A.
Clinic for General Internal Medicine and Psychosomatics
Heidelberg University
Thibautstrasse 4
D-69115 Heidelberg

Phone number +49 (0) 6221 - 56 - 38 39 6
Fax +49 (0) 6221 - 56 – 53 30
markus.haun@med.uni-heidelberg.de

Biometrician

Prof. Dr. sc. hum. Dipl.-Psych. Dipl.-Math. Beate Wild
Head Biometrician
Clinic for General Internal Medicine and Psychosomatics
Heidelberg University
Im Neuenheimer Feld 410
D-69120 Heidelberg

Funding

Central Research Institute of Ambulatory Health Care in Germany
Foundation of civil law, Salzufer 8, 10587 Berlin

Date and Version of the protocol

Date: 04.05.2022, Version: 2.0

Signature of Study Director

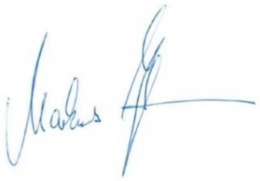

©EKHD-Struktur-Protokoll-Sonstige-Studien-DO-017-211209

1. Summary

Background

Patients who have been treated for depressive or anxiety disorders at a mental healthcare ward often do not find their way to the urgently indicated outpatient follow-up treatment after discharge. This break in the continuity of care causes significantly high rehospitalization rates both very shortly after discharge and in the further course. This lack of continuity of care can lead to the disorder becoming chronic and to decreased social participation (e.g. in the family or on the labor market) or, in extreme cases, a complete lack thereof.

Aim of Study

A Care Transition Intervention (CTI) will be tested for feasibility under study conditions. The aim of this intervention is to enable a smoother transition between the inpatient and outpatient sector of psychosocial care for patients and thus to avoid rehospitalizations, to ensure social participation in the medium term and to improve the quality of life. In the course of a feasibility study, it will be exploratively examined whether the implementation of the CTI is possible and shows the potential to realize the above-mentioned goals, i.e. whether it can ultimately contribute to an improvement in care.

Study design and implementation

An individual randomized controlled feasibility study will be conducted. The CTI is aimed at patients 18 years of age or older with clinically significant depression and anxiety at the transition from inpatient to outpatient psychosocial care. A total of 50 subjects will be enrolled in the study and randomized in a 1:1 ratio into two groups. For pre-post evaluation, questionnaire instruments as well as qualitative interviews will be used. The analysis will be descriptive for the quantitative endpoints and software-based content analysis for the qualitative data. The study will be registered in a study registry before inclusion of the first patient.

2. Inhaltsverzeichnis

1. Summary **Fehler! Textmarke nicht definiert.**
2. Table of contents 3
3. Introduction/Scientific basis 5
4. Aims of the study (general) 6
5. Target criteria 6
   5.1 Main target criteria 6
   5.2 Secondary target criteria 7
6. Study-related measures 7
   6.1 Recruitment and clarification **Fehler! Textmarke nicht definiert.**
   6.2 Data collection 8
   6.3 Treatment conditions 9
   6.4 Study process 12
   6.5 Quality assurance measures 13
7. Expected benefit 13
8. Potential risks or burdens 14
9. Study type/design 14
10. Inclusion and exclusion criteria 15
    10.1 Inclusion criteria 15
    10.2 Exclusion criteria 15
11. Randomization procedure/plan 15
12. Termination criteria 16
    12.1 Individual termination criteria 16
    12.2 Termination criteria for the study as a whole 16
13. Statistical design 16
    13.1 Statistical methods 16
    13.2 Caseload planning 17

14. Legal and ethical aspects 17
14.1 Declaration of Helsinki 17
14.2 Review by the Ethics Committee 17
14.3 Information on the voluntary nature of participation 17
14.4 Information on education and consent 18
14.5 Information on the right of withdrawal and data destruction in the event of withdrawal 18
14.6 Data protection information 18
14.7 Details of a permit according to StrlSchG 18
14.8 Funding/institutional links/conflicts of interest 18
14.9 Information on insurance (commuting accident/StrlSchV) 19
15. Bibliography 7
16. Attachments 22

3. Introduction / scientific basis

Patients who have been treated as inpatients for a depressive disorder and/or anxiety disorder usually do not find their way to the urgently indicated outpatient follow-up treatment after discharge from inpatient treatment.[1–4] **Failure to provide this outpatient treatment leads to high rates of rehospitalization, reduced medication adherence, an increased risk of chronification of the respective disorder and reduced participation in the labor market.** [4–7] **Reasons for not starting outpatient follow-up treatment can be found on several levels and range from structural barriers such as a low availability of treatment places to barriers on the individual level such as ambivalent motivation towards further treatment, lack of access to information about available treatment o**ptions **and resulting difficulties to orientate oneself in the health care system.**[8] **The barriers at the individual level of the patients appear to be particularly obstructive, but at the same time they can be modified comparatively quickly**. [9] **It has been shown that patients often need support to prepare for the time after their inpatient stay and to find their way in the complex system of outpatient psychosocial care. This is precisely the point at which so-called Care Transition Interventions (CTI) come into play, guaranteeing close support for these patients at an early stage, i.e. before discharge, and for a certain time after discharge. This involves mediation between different service providers (integrated care) and patients should be enabled to deal with their disorder in more detail and to seek further support independently (e.g. according to the approach of patient emancipation through psychoeducation). The aim of CTI is to enable patients to make an uninterrupted transition between the inpatient and outpatient sectors of psychosocial care and thus to ensure social participation (e.g. in the family or at work) and to avoid rehospitalization.**[10] In order to improve the transition from inpatient to outpatient psychosocial care, the German health care system has so far adopted approaches that focus on fixed institutional cooperation between service providers, which in turn should promote treatment continuity[11].These approaches offer the advantage of building sustainable networks of care providers in the respective region, allowing patients to be referred more quickly, which in turn leads to symptom improvements, quality-of-life gains, and increased medication adherence.[12] The difficulty of more complex cooperation between different participants (e.g., service providers, health insurers, associations) is shown by the fact that some promising projects have been stopped and discontinued [11]. For this reason,

the use of low-threshold CTI seems to make sense, in which work is done individually with the patients at the interface to the respective outpatient services that are available. Results from reviews of CTI show that there is great heterogeneity in terms of intervention design, target group, and outcome variables[10,13,14] For example, the spectrum of interventions ranges from support during the discharge process by peers and/or social workers to continued treatment by the ward physician even after the end of the inpatient stay. Nevertheless, CTI have proven to be effective and, especially in the field of psychosomatic medicine and psychotherapy, additionally cost-efficient.[15] In particular, they reduce rehospitalizations and improve both the individual quality of life of patients and their re-entry into the labor market compared to patients who do not receive CTI in standard care [10,16,17] Since these promising results come mainly from the United States, where the boundaries between the inpatient and outpatient sectors are more flexible and less rigid than in the German health care system, it is appropriate to test CTI in Germany as well in order to address the problems of intersectoral care.

4. Ziele der Studie (allgemein)

Ziel des beschriebenen Forschungsprojekts ist die Prüfung der Machbarkeit einer CTI zur niederschwelligen Unterstützung von Patient*innen mit depressiven Störungen und Angststörungen beim Übergang von stationärer zu ambulanter psychosozialer Versorgung. Dabei soll untersucht werden, ob sowohl Patient*innen als auch Leistungserbringer die Intervention akzeptieren und diese umgesetzt werden kann. Eine Nebenfragestellung zielt auf die Passung einzelner vorher festgelegter Interventionskomponenten ab. Ergebnisse dieses Projekts können dafür genutzt werden, die Intervention weiterzuentwickeln und hinsichtlich der Bedarfe der Patient*innen zu optimieren. Im Anschluss kann die Effektivität in einer ausreichend gepowerten randomisiert-kontrollierten Studie überprüft werden.

5. Zielkriterien

5.1 Hauptzielkriterien[18,19]

- Machbarkeit der Rekrutierung: Etablierung suffizienter Rekrutierungswege für Interventions- und Kontrollgruppe und Bestimmung der erforderlichen Ressourcen
  Operationalisierung über Recruitment Yield
- Machbarkeit der Randomisierung: Akzeptanz bei Patient*innen und Behandler*innen

Operationalisierung über Consent Rate

- Angemessenheit (Appropriateness) und Machbarkeit der Intervention: Praktikabilität der Abläufe sowie Adhärenz von Proband*innen sowie des*der CTN
  Operationalisierung über Haltequote für beide Studienarme (Zahl der jeweils von den Proband*innen wahrgenommenen Interventionstermine), über quantitative und qualitative Erfassung von unerwünschten Ereignissen bzw. Effekten sowie über quantitative (regelmäßige systematische Abfrage zu einzelnen Interventionskomponenten) und qualitative Erfassung der Umsetzung (logistische und technische Realisierung der Intervention v.a. hinsichtlich Terminierung, Umsetzung der Intervention inkl. deren Dokumentation, Umsetzungstreue und Sicherheit der Patient*innen)
- Machbarkeit der Datenerhebung: Eignung der Instrumente zur Outcome-Messung (Responsiveness der Instrumente und Anteil der fehlenden Daten) und effektive Verblindung der Endpunkterheber*innen
  Operationalisierung über Exploration von Boden- und Deckeneffekte für die quantitativen Maße sowie über Bestimmung der Loss to Follow-up Raten und Zahl der ungeplanten vorzeitigen Entblindungen

5.2 Nebenzielkriterien

- Wiedereinweisungsquote (Rehospitalisierung)
- Inanspruchnahme psychosozialer Behandlungsangebote (Fragebogen zur Inanspruchnahme medizinischer und nicht medizinischer Versorgungsleistungen bei psychischen Erkrankungen, FIMPsy) [20]
- Psychische Symptomlast (Depressivität, Ängstlichkeit) (Patient Health Questionnaire Anxiety and Depression Scale, PHQ-ADS) [21]
- Lebensqualität (12-Item Short-Form Health Survey, SF-12) [22]
- Recovery (Recovery Assessment Scale, RAS-G) [23]

6. Studienbedingte Maßnahmen

Recruitment and education

Patients are recruited for AMBITION during their inpatient stay at the Clinic for General Internal Medicine and Psychosomatics at Heidelberg University Hospital. Suitable patients will be selected (approximately) from the middle of their treatment period (usually corresponding to the fifth week of treatment) by their reference therapist in the course of individual psychotherapeutic consultations, informed about the study and provided with the study documents, consisting of information leaflet, consent form in duplicate and the baseline questionnaire set. If the

patient agrees in writing by signature to receive further information, he/she will be contacted by telephone by the study center within two days. The patient will be (1) assessed for compliance with inclusion and exclusion criteria, (2) provided with detailed information about the study if eligible, and (3) given ample opportunity to ask questions. Steps 1 and 2 will take no longer than 15 minutes. If study inclusion is not possible, this will be documented with reason for refusal, age and gender. After the informed consent interview by the study center, the patients interested in participating in the study complete the baseline questionnaire, sign the informed consent form and hand in the study documents at the team base of the respective ward. Subsequently, randomization and allocation of the subjects to the two study arms takes place within 48 hours. The CTN will inform the subjects of the result of the allocation by telephone. In the case of subjects who are assigned to the intervention condition (CTI), a first appointment with the CTN is already made during this telephone call. The CTN communicates the result of the randomization to the respective reference therapist.

6.1 Data collection

Surveys of the test persons

Patients participating in the study will receive a questionnaire set consisting of the following validated questionnaires in German version: Patient Health Questionnaire Anxiety and Depression Scale (PHQ-ADS),[21] 12-item Short-Form Health Survey (SF-12)[22] and Recovery Assessment Scale (RAS-G).[23] In addition, the Questionnaire on Utilization of Medical and Non-Medical Care Services for Mental Illness (FIMPsy) is used to obtain data on the use of health care services of any kind and rehospitalizations.[20] Patients complete these questionnaires for the first time before randomization on the ward (either as a paper version sent by mail or online via Unipark (Tivian XI GmbH)). The post-survey takes place three months and two further follow up surveys six and nine months after inclusion in the study and are conducted by a member of the study team who is blinded to the group membership of the subjects (blinded endpoint survey). In order to gain a more differentiated picture of the subjects' post-inpatient utilization, the use of all possible outpatient psychosocial care services is queried during the post and follow-up surveys. Answering the questionnaires takes about 15 to 20 minutes each (empirical value from similar previous studies, e.g. PROVIDE-B).[24,25] The latest possible date for the poster survey or follow up surveys is 14 days after the actual scheduled date (three, six or nine months after inclusion). After the post-survey, qualitative interviews on the feasibility of the intervention will be conducted with patients* in the intervention condition (see interview guide in Appendix A). These interviews will last approximately 30 minutes and will be recorded for

analysis and transcribed as soon as possible . The audio/video recordings will be deleted afterwards.

Surveys of the Care Transition Navigator (CTN).*

At the end of the intervention, an interview is conducted with the CTN. The interview will focus primarily on the topics of appropriateness, feasibility and anticipated benefits of the intervention. Of particular interest are the experiences and insights of the CTN during the intervention implementation (see interview guide in Appendix B). Participation in the interview requires Informed Consent including written consent from the* CTN. The interview will last approximately 30 minutes and will be recorded for analysis and transcribed as soon as possible. The audio/video recordings will be deleted afterwards.

6.3 Treatment conditions

Intervention condition

The intervention whose feasibility is to be tested is composed of core components and optional components. Core components are parts of the intervention that have to be applied compulsorily. The optional components are used as needed, which means that the test person can be individually accompanied. The design of the outpatient psychosocial follow-up therapy is primarily based on the recommendations of the attending ward physicians and psychotherapists. The first core component is the identification and immediate subsequent addressing of barriers, , which prevent the test person from taking up outpatient psychosocial follow-up treatment . Motivational interviewing and psychoeducation should motivate the patient to make and keep appointments with psychosocial contact points after the stay in the ward. The test person is supervised by a CTN, whereby this is an organizational support of the test person and explicitly not a psychotherapeutic activity. In preparation for the implementation of the intervention, the CTN will receive extensive study-related training in motivational interviewing and social work topics (core component 2). The* CTN will be a research assistant with a bachelor's degree in psychology. The*subject, guided by the CTN, will develop a subjective summary of the ward stay. This enables the CTN to recognize the weighting of challenges but also of what has been achieved so far from the subject's point of view and to address them more precisely in further joint work (core component 3). For example, the outpatient therapist can be selected on the basis of factors that have already contributed to the improvement of the respective test person during the inpatient stay. These steps are taken before the patient is discharged.

After discharge, further appointments take place between the test person and the CTN, in which barriers on the test person's side are continuously identified and addressed and, if

necessary, the ambivalence towards follow-up treatment is reflected with the test person with the aim of building up motivation. In the first month after discharge, the appointments between the test person and the CTN take place at least every two weeks; in months two and three after discharge, at least one appointment per month takes place. In addition to the core components, some of the optional components will also be part of the appointments between the proband and the CTN. If there is a need on the part of the subject for more structuring of the intervention, a patient-centered written brief discharge plan will be developed jointly by the subject and the CTN. This should include the following points: (1) clarification of the need and (2) the design of a follow-up treatment, (3) identification of possible barriers to taking up this treatment, (4) offer of where support can be provided, (5) agreement on how often and to what extent appointments between the proband and the CTN will take place within the first three months after discharge (1st month at least every 2 weeks; 2nd and 3rd month at least one appointment per month). In order to support and motivate the test person, the CTN can look at the range of psychosocial therapists in the vicinity of the test person's home. Furthermore, the patient should be motivated to contact the family doctor after discharge and to inform him/her about the further course of treatment. Other components of support and motivation may vary from individual to individual depending on the severity of stress and resources of the respondent, but will include components of the following spectrum: systematic reminders of and follow-up on appointments and medication, screening for suicidality, evaluation of known and/or emerging barriers, psychoeducation on dealing with mental disorder and coping strategies, and behavioral activation (maintaining a positive attitude, solution orientation, encouraging self-help). These optional components have been shown to be effective in several similar studies.[10,26] If a face-to-face meeting is not possible, appointments between the respondent* and CTN will be conducted via video consultation. It is planned that the appointments between subject and CTN will last between 30 and 60 minutes.

In addition, the CTN will attend a case supervision session led by a senior physician every two weeks, , in which all probands will be discussed. In particular, it will be discussed how to deal with hard-to-reach and less adherent probands in order to successfully implement the transition to outpatient psychosocial follow-up treatment in this group as well (core component 4).

Tabular intervention description

Core components	Effect/Benefit
**Barrier management**	Identify/address barriers to preparing for follow-up treatment.
**Supervision by trained Care Transition Navigator**	Expertise in common problem areas, support in navigating the treatment system.
**Reflection of the station stay**	Focusing on aspects perceived as important from the respondent's point of view
**Supervision**	Quality assurance of care for subjects who are difficult to cooperate with.
**Optional components**	**Effect/Benefit**
**Written short layoff plan**	Joint development of aspects to be focused on
**Systematic remembering**	Support for treatment continuity
**Screening for suicidality,**	Timely recognition of self-harming behavior
**Psychoeducation regarding coping with mental disorder.**	Sustainable independent handling of possibly chronic stresses
**Behavioral Activation**	Maintaining a positive attitude, solution orientation, stimulation of self-help

Control condition

Subjects in the control condition are supported in their search for outpatient psychosocial follow-up treatment as determined by the reference therapist (e.g. by being given contact details of outpatient psychotherapists in the vicinity and motivated to make contact during the further course of inpatient treatment). This can also - but does not have to - mean admission to the ward's aftercare group. Within the framework of this offer, meetings are held once a week in a group format over a period of five weeks after discharge in order to discuss problems in organizing follow-up treatment and to provide general and less individualized support.

6.4 Course of studies

Schematic schedule (according to SPIRIT 2013 Statement)

			STUDY SECTION							
	**Inclusion**	**Alloca-tion**	**Postal allotment**					**follow-up**		**Close-out**
**TIME**	t_0	t_1	t_{1a}	t_{1b}	t_{1c}	t_{1d}	t_2 (t_1 +3 months)	t_3 (t_1 +6 months)	t_4 (t_1 +9 months)	t_x
INCLUSIONS:										
Screening for inclusion and exclusion criteria	X									
Informed Consent	X									
Baseline survey	X									
Randomization		X								
Allocation		X								
INTERVENTIONS:										
Care Transition Inter-vention (CTI)			←	—	—	→				
Treatment as usual			←	—	—	→				
EARNINGS:										
Sociodemographics	X									
PHQ-ADS, SF-12, RAS-G, FIMPsy	X						X	X	X	
*Interviews: CTN and patient*s from the Intervention Condition*							X			

Patients who are eligible for participation in the study will be approached about the study during their inpatient stay in the course of individual psychotherapy and provided with the study documents. In the case of written informed consent, patients will be contacted by the study team within two days in order to be assessed with regard to the inclusion and exclusion criteria and to receive detailed information about the study (t_0 in the schematic schedule). As soon as the signed Informed Consent and the completed baseline questionnaire have been submitted to the study team, randomization and allocation of patients will take place within a further two days (t_1). The subsequent intervention period is three months (t_{1a-d} representative of four CTI appointments). Subjects in the intervention condition will receive the Care Transition intervention through the CTN. Subjects in the control condition will be supported in the course of inpatient treatment by the reference therapist and, if necessary, post-inpatient as part of the aftercare group in the search for outpatient psychosocial follow-up treatment. The post-survey will be conducted three months after study inclusion (t_2). Two quantitative follow-up surveys are planned six (t_3) and nine months (t_4) after inclusion. Qualitative interviews with the CTN and subjects in the intervention condition will be conducted at the end of the intervention period (CTN) or following the poster survey (t_2) (subjects).

6.5 Quality assurance measures

- Adherence check: After each intervention session, the CTN will complete an online questionnaire asking which intervention components were used during the session. In addition, the CTN is required to maintain detailed intervention documentation throughout the intervention.
- Supervision: The CTN will be supervised during the study by a senior physician of the Department of General Internal Medicine and Psychosomatics. The supervision takes place every 14 days.
- Data management: To minimize data entry errors, the input fields are programmed so that only valid values can be entered. Entered data sets are continuously checked for consistency with previously entered data. Data cleaning is performed independently by two members of the study team.

7. Expected benefit

A CTI can help increase patients' health-related quality of life while reducing rehospitalizations of recently discharged mental health patients[10]. Specifically, this means that subjects in the intervention condition are less likely to need to be readmitted to inpatient treatment due to

additional support in organizing outpatient psychosocial follow-up treatment and that symptom improvement results from continuous treatment. Subjects in the control condition do not receive any additional support, but no measures outside the study are excluded.

On a societal level Analyses of the cost-effectiveness of comparable interventions come to different conclusions. In one study, the costs are considered too high for a gain in quality of life.[28] In contrast, another more intensive CTI not only showed a positive clinical effect, but was also more cost-effective than standard care.[15] In the presented project, the feasibility of a CTI should be investigated, in order to subsequently test the efficacy and efficiency in a larger randomized-controlled intervention study with health economic evaluation (piggy-back design) if feasibility is given. In order to avoid unnecessary costs with regard to this potential large confirmatory study, to design the intervention as efficiently as possible and to align it as best as possible with the needs of the patients, it is important to test the feasibility and acceptance of the intervention in advance in the course of a feasibility study.

8. Possible risks or burdens

It is not to be assumed that the use of the described CTI is associated with risks and/or disadvantages for the test persons, since the intervention provides for additional support in the search for outpatient psychosocial follow-up treatment and no components of routine care are curtailed as a result. Only the sporadic preoccupation with possibly problematic life contents, which is provided for in the intervention at best, can sometimes be accompanied by temporary self-doubt, interim symptom deterioration and changes in personal relationships. These are natural reactions and not a sign of an unfavorable course. However, given the relatively low intensity of the intervention, these risks are unlikely. The completion of the instruments in the context of the survey of the main and secondary target criteria will not exceed 20 minutes each for baseline and poster survey.

9. Study type/design

This is a monocentric, prospective, endpoint collector-blinded and individually randomized controlled feasibility study with qualitative process evaluation (interviews with subjects and the CTN). Patients with depression and/or anxiety disorders treated as inpatients at the Department of General Internal Medicine and Psychosomatics at Heidelberg University Hospital are supported by a CTN during the transition to the outpatient sector of psychosocial care. The intervention period is three months after discharge from the psychotherapeutic ward. Data collections will occur at inclusion in the study prior to randomization (baseline survey) and three (poster survey), six, and nine months (follow up surveys) after discharge. As the focus is on feasibility testing, this is a descriptive study without hypothesis testing.

10. Inclusion and exclusion criteria

10.1 Inclusion criteria for patients

- Recommendation of the attending ward physician and/or psychotherapist to start outpatient psychosocial follow-up treatment.
- completed 18th year of life
- inpatient treatment for at least moderate depression and/or anxiety disorder at Heidelberg University Hospital
- Informed Consent documented in writing.
- Capacity to consent

10.2 Exclusion criteria for patients

- Planned resumption of outpatient guideline psychotherapy (short-term or long-term therapy) already started prior to the current inpatient stay after inpatient stay
- active suicidal thoughts or active thoughts of doing violence to others
- acute psychotic states
- Dementia and/or severe cognitive impairment
- insufficient German language skills

10.3 Inclusion criteria for the* CTN

- Professional degree in one of the following professional fields: Medicine, psychology, nursing, social sciences, humanities and linguistics, social work.
- excellent German language skills
- Informed Consent documented in writing.

10.4 Exclusion criteria for the* CTN

- No age of majority

11. Randomization procedure/plan

Patients will be randomly assigned to one of the two study conditions (CTN support vs. treatment-as-usual, TAU, i.e., usual discharge management without specific additional support in finding follow-up therapy) in a 1:1 ratio. Randomization will be performed independently by the study center (Stephanie Estel, Department of General Internal Medicine and Psychosomatics,

Heidelberg University) after obtaining informed consent and no later than 28 days after the last data collection (screening or baseline). The web-based program *Randomizer version 2.1.0 of* the Institute of Medical Informatics, Statistics and Documentation of the Medical University of Graz (https://www.randomizer.at) is used, which ensures confidentiality of the treatment sequence until allocation by central randomization. The treatment sequence is generated by a computer-generated sequence of random numbers. Block randomization with stratification of the patients according to baseline values in the PHQ-ADS (depressiveness and anxiety) is used.

12. Termination criteria

12.1 Individual termination criteria

The withdrawal of the subject's consent to participate in the study is defined as a criterion for termination, whereby consent to participate in the study can be withdrawn at any time and without giving reasons. In case of withdrawal from the study, the subjects will be asked whether they agree with the analysis of the data. If they do not agree, the data will be destroyed.

12.2 Termination criteria for the study as a whole

None.

13. Statistical design

13.1 Statistical methods

This is a descriptive study whose primary objective is to test and determine the feasibility of a CTI. The feasibility and acceptability of the intervention and study procedures will be assessed using the criteria outlined above. Baseline post-comparison of the above secondary evaluation content among subjects will be primarily descriptive with determination of central location measures (mean, median) and presentation of variability measures (standard deviation, interquartile range, and range) as well as absolute and relative frequencies. Following a descriptive processing, difference tendencies in the above mentioned evaluation contents (dependent variables) between intervention and control condition (independent variable) are to be determined. For this purpose, mean differences and effect sizes (according to Cohen) will be calculated together with the respective 95% confidence intervals. Since the study is purely exploratory in nature, the analysis has no confirmatory value. The analysis will also include a closer look at the non-responders and refusers (including the reasons for refusal) compared to the included

subjects. Analogous statistical methods will be used for group comparison. If subjects drop out of the study, a separate description of this sample will be provided in order to gain indications of possible selectivity. All statistical analysis will be done in R (version 4.1.0 or higher). The study will be entered into a study registry (WHO International Clinical Trials Registry) prior to inclusion of the first subject.

13.2 Caseload planning

The sample size is 50 subjects who will be randomly assigned in a 1:1 ratio to either the intervention condition, which includes CTI, or the control condition, which includes usual care following an inpatient stay. Because the requested study is a feasibility study that is purely exploratory in nature and does not include confirmatory analyses, no endpoint-driven case number planning was performed. A sample size of 50 subjects was used as a guideline for studies that primarily test study processes for feasibility.[31] Taking into account the number of discharged patients from the respective wards, a recruitment period of three months is expected.

14. Legal and ethical aspects

14.1 Declaration of Helsinki

The examination will be performed in accordance with the Declaration of Helsinki and the Professional Code of Conduct for Physicians of the State Medical Association of Baden-Württemberg in the respective current versions. The ICH-GCP guidelines will be taken into account.

14.2 Review by the Ethics Committee

The study protocol will be submitted to the Ethics Committee of the Medical Faculty of Heidelberg for review prior to the start of the study. The inclusion of subjects will not commence until the Ethics Committee has given its written approval.

14.3 Information on the voluntary nature of participation

The participation of the test persons is voluntary.

14.4 Informed consent details

Before the start of the study, the participants will be informed verbally and in writing about the nature and scope of the planned study, in particular about the possible benefits for their health and any risks. Their consent will be documented by signing the consent form.

14.5 Information on the right of withdrawal and data destruction in the event of withdrawal

The consent can be withdrawn by the subject at any time, without giving reasons and without disadvantages for further medical care.
If the subject withdraws from the study, he/she will be asked whether he/she agrees with the analysis of the data. If he/she does not agree, the data will be destroyed. If no clarification is possible, data already collected will be destroyed.

14.6 Data protection information

The names of the test persons and all other confidential information are subject to medical confidentiality and the provisions of the General Data Protection Regulation (DSGVO) and the State or Federal Data Protection Act (LDSG or BDSG). If necessary, data of the test persons will only be passed on in pseudonymized form. The pseudonymization of the data already takes place before the questionnaires are issued. The pseudonymization key is managed confidentially by the study director and remains with him. Third parties do not gain insight into original documents. In publications, e.g. in scientific journals, no reference to individual persons can and will be made. The data collected or obtained in the study will be stored in accordance with the applicable guidelines for up to ten years after the date of the survey and then destroyed. As soon as possible, but at the latest after completion of all publications, the personal data will be anonymized in accordance with § 35 para. 2 LDSG BW.

14.7 Information on a permit according to StrlSchG

Not applicable.

14.8 Funding/institutional links/conflicts of interest

The study is sponsored by the Central Institute for Health Care in the Federal Republic of Germany, a foundation under civil law. There are no conflicts of interest between the members of the study team and third parties.

14.9 Information on insurance (commuting accident/StrlSchV)

Not required.

15. Literaturverzeichnis

1. Jørgensen K, Bonde Dahl M, Frederiksen J. Healthcare Professionals' and Users' Experiences of Intersectoral Care between Hospital and Community Mental Healthcare. *Int J Environ Res Public Health*. 2020;17(18). doi:10.3390/ijerph17186510

2. Ko Y, Park S. Life after hospital discharge for people with long-term mental disorders in South Korea: Focusing on the "revolving door phenomenon." *Perspectives in Psychiatric Care*. 2021;57(2):531-538. doi:https://doi.org/10.1111/ppc.12575

3. Neiterman E, Wodchis WP, Bourgeault IL. Experiences of Older Adults in Transition from Hospital to Community*. *Canadian Journal on Aging / La Revue canadienne du vieillissement*. 2015;34(1):90-99. doi:10.1017/S0714980814000518

4. Wiegand HF, Saam J, Marschall U, et al. Challenges in the Transition from In-Patient to Out-Patient Treatment in Depression. *Deutsches Aerzteblatt Online*. Published online July 6, 2020. doi:10.3238/arztebl.2020.0472

5. Niimura J, Tanoue M, Nakanishi M. Challenges following discharge from acute psychiatric inpatient care in Japan: patients' perspectives. *Journal of Psychiatric and Mental Health Nursing*. 2016;23(9-10):576-584. doi:https://doi.org/10.1111/jpm.12341

6. Olfson M, Marcus SC, Doshi JA. Continuity of Care After Inpatient Discharge of Patients With Schizophrenia in the Medicaid Program: A Retrospective Longitudinal Cohort Analysis. *J Clin Psychiatry*. 2010;71(7):0-0. doi:10.4088/JCP.10m05969yel

7. Wright N, Rowley E, Chopra A, Gregoriou K, Waring J. From admission to discharge in mental health services: a qualitative analysis of service user involvement. *Health Expectations*. 2016;19(2):367-376. doi:https://doi.org/10.1111/hex.12361

8. Storm M, Husebø AML, Thomas EC, Elwyn G, Zisman-Ilani Y. Coordinating Mental Health Services for People with Serious Mental Illness: A Scoping Review of Transitions from Psychiatric Hospital to Community. *Adm Policy Ment Health*. 2019;46(3):352-367. doi:10.1007/s10488-018-00918-7

9. Nolan P, Bradley E, Brimblecombe N. Disengaging from acute inpatient psychiatric care: a description of service users' experiences and views. *Journal of Psychiatric and Mental Health Nursing*. 2011;18(4):359-367. doi:10.1111/j.1365-2850.2010.01675.x

10. Hegedüs A, Kozel B, Richter D, Behrens J. Effectiveness of Transitional Interventions in Improving Patient Outcomes and Service Use After Discharge From Psychiatric Inpatient Care: A Systematic Review and Meta-Analysis. *Front Psychiatry*. 2020;10. doi:10.3389/fpsyt.2019.00969

11. Schmid P, Steinert T, Borbé R. Systematische Literaturübersicht zur Implementierung der sektorübergreifenden Versorgung (Regionalbudget, integrierte Versorgung) in Deutschland. *Psychiatr Prax*. 2013;40(8):414-424. doi:10.1055/s-0033-1343192

12. Lambert M, Bock T, Schöttle D, et al. Assertive Community Treatment as Part of Integrated Care Versus Standard Care: A 12-Month Trial in Patients With First- and Multiple-Episode Schizophrenia Spectrum Disorders Treated With Quetiapine Immediate Release (ACCESS Trial). *J Clin Psychiatry*. 2010;71(10):0-0. doi:10.4088/JCP.09m05113yel

13. Gaebel W, Kerst A, Janssen B, et al. EPA guidance on the quality of mental health services: A systematic meta-review and update of recommendations focusing on care coordination. *Eur Psychiatry*. 2020;63(1). doi:10.1192/j.eurpsy.2020.75

14. Vigod SN, Kurdyak PA, Dennis CL, et al. Transitional interventions to reduce early psychiatric readmissions in adults: systematic review. *The British Journal of Psychiatry*. 2013;202(3):187-194. doi:10.1192/bjp.bp.112.115030

15. Moessner M, Bauer S, Özer F, Wolf M, Zimmer B, Kordy H. Cost-effectiveness of an Internet-based aftercare intervention after inpatient treatment in a psychosomatic hospital. *Psychotherapy Research*. 2014;24(4):496-503. doi:10.1080/10503307.2013.845919

16. Jackson C, DuBard A, Swartz M, et al. Readmission Patterns and Effectiveness of Transitional Care Among Medicaid Patients With Schizophrenia and Medical Comorbidity. *North Carolina Medical Journal*. 2015;76(4):219-226. doi:10.18043/ncm.76.4.219

17. Reynolds W, Lauder W, Sharkey S, Maciver S, Veitch T, Cameron D. The effects of a transitional discharge model for psychiatric patients. *Journal of Psychiatric and Mental Health Nursing*. 2004;11(1):82-88. doi:https://doi.org/10.1111/j.1365-2850.2004.00692.x

18. Proctor E, Silmere H, Raghavan R, et al. Outcomes for Implementation Research: Conceptual Distinctions, Measurement Challenges, and Research Agenda. *Adm Policy Ment Health*. 2011;38(2):65-76. doi:10.1007/s10488-010-0319-7

19. Eldridge SM, Lancaster GA, Campbell MJ, et al. Defining Feasibility and Pilot Studies in Preparation for Randomised Controlled Trials: Development of a Conceptual Framework. *PLOS ONE*. 2016;11(3):e0150205. doi:10.1371/journal.pone.0150205

20. Grupp H, König HH, Riedel-Heller S, Konnopka A. FIMPsy – Fragebogen zur Inanspruchnahme medizinischer und nicht medizinischer Versorgungsleistungen bei psychischen Erkrankungen: Entwicklung und Verwendung. *Psychiatr Prax*. 2018;45(02):87-94. doi:10.1055/s-0042-118033

21. Kroenke K, Spitzer RL, Williams JBW, Löwe B. The Patient Health Questionnaire Somatic, Anxiety, and Depressive Symptom Scales: a systematic review. *General Hospital Psychiatry*. 2010;32(4):345-359. doi:10.1016/j.genhosppsych.2010.03.006

22. Ware JE, Kosinski M, Keller SD. A 12-Item Short-Form Health Survey: Construction of Scales and Preliminary Tests of Reliability and Validity. *Medical Care*. 1996;34(3):220-233.

23. Cavelti M, Wirtz M, Corrigan P, Vauth R. Recovery assessment scale: Examining the factor structure of the German version (RAS-G) in people with schizophrenia spectrum disorders. *Eur Psychiatry*. 2017;41:60-67. doi:10.1016/j.eurpsy.2016.10.006

24. Tönnies J, Hartmann M, Wensing M, et al. Mental health specialist video consultations for patients with depression or anxiety disorders in primary care: protocol for a randomised controlled feasibility trial. *BMJ Open*. 2019;9(9). doi:10.1136/bmjopen-2019-030003

25. Tönnies J, Hartmann M, Wensing M, et al. Mental Health Specialist Video Consultations Versus Treatment-as-Usual for Patients With Depression or Anxiety Disorders in Primary Care: Randomized Controlled Feasibility Trial. *JMIR Mental Health*. 2021;8(3):e22569. doi:10.2196/22569

26. Steffen S, Kösters M, Becker T, Puschner B. Discharge planning in mental health care: a systematic review of the recent literature. *Acta Psychiatrica Scandinavica*. 2009;120(1):1-9. doi:10.1111/j.1600-0447.2009.01373.x

27. Abu HO, Anatchkova MD, Erskine NA, et al. Are we “missing the big picture” in Transitions of Care? Perspectives of Healthcare Providers Managing Patients with Unplanned Hospitalization. *Appl Nurs Res*. 2018;44:60-66. doi:10.1016/j.apnr.2018.09.006

28. Puschner B, Baumgartner I, Loos S, et al. Kosteneffektivität bedarfsorientierter Entlassungsplanung bei Menschen mit hoher Inanspruchnahme psychiatrischer Versorgung. *Psychiatr Prax*. 2012;39(08):381-387. doi:10.1055/s-0032-1327188

29. Jacobi F, Höfler M, Siegert J, et al. Twelve-month prevalence, comorbidity and correlates of mental disorders in Germany: the Mental Health Module of the German Health Interview and Examination Survey for Adults (DEGS1-MH). *Int J Methods Psychiatr Res*. 2014;23(3):304-319. doi:10.1002/mpr.1439

30. Wittchen HU, Jacobi F. *Angststörungen*. Nachdr. Robert Koch-Inst; 2007.

31. National Insitute for Health Research. Justify sample size for a feasibility study. RDS London. Accessed June 28, 2021. https://www.rds-london.nihr.ac.uk/resources/justify-sample-size-for-a-feasibility-study/

16. Anhänge

Anhang A. Interviewleitfaden für Interviews mit Patient*innen zu t_2

A. Einführung und Projektvorstellung

- Vorstellen Interviewer*in und Protokollant*in
- Hinweis auf Audio- und Videoaufzeichnung, Datenschutz, Einholung schriftlicher Informed Consent
- Schilderung des Interviewablaufs
- Einholung demographischer Informationen: Alter, Schulabschluss, Partnerschaft

B. Inhaltliche Fragen

- Wie zufrieden sind Sie aktuell mit Ihrer gesundheitlichen Situation?
- Wie zufrieden sind Sie mit der Unterstützung durch die/den Care Transition Navigator?
- Wie haben Sie die Kommunikation zwischen Ihnen und der/dem Care Transition Navigator?
- Wie patientenfreundlich haben Sie die Unterstützung nach diesem Ansatz erlebt?
- Würden Sie anderen diese Art der Unterstützung empfehlen?
- Wie empfanden Sie die Beziehung mit der der/dem Care Transition Navigator?
- Wie beurteilen Sie den Zugang zu psychosozialer Unterstützung im Lichte der Care Transition Intervention?
- Was an der Unterstützung durch die/den Care Transition Navigator war für Sie besonders hilfreich?
- Gibt es etwas, dass Sie in der Umsetzung anders machen würden?

C. Abschluss

- Gibt es Aspekte, die Ihnen noch wichtig sind und wir noch nicht thematisiert haben? Haben Sie noch Fragen?

Anhang B. Interviewleitfaden für Interviews mit der/dem Care Transition Navigator zu t_2

A. Einführung und Projektvorstellung

- Vorstellen Interviewer*in und Protokollant*in
- Hinweis auf Audio- und Videoaufzeichnung, Datenschutz, Einholung schriftlicher Informed Consent
- Schilderung des Interviewablaufs
- Einholung demographischer Informationen: Alter, Schulabschluss, Partnerschaft

B. Inhaltliche Fragen

- Wenn Sie an Ihre positiven und negativen Erfahrungen mit der Care Transition Intervention denken, wie sieht Ihre Bilanz aus?
- Wie effektiv haben Sie die Unterstützung für die Patientinnen und Patienten erlebt?
- Welche Rückmeldungen haben Sie von den Patientinnen und Patienten erhalten?
- Wie haben Sie den Übergang aus der stationären Behandlung der Patientinnen und Patienten erlebt?
- Wie praktikabel war die Terminfindung?
- Wie beurteilen Sie die Begegnung mit Patientinnen per Videokonsultation? (wenn stattgefunden)
- Wie zufrieden sind Sie mit dem Management von Notfällen?
- Wie zufrieden waren Sie mit dem Umfang, in dem Sie dem Studienteam Feedback geben konnten?
- Inwiefern haben Sie Verbesserungen in der Motivation und Eigenständigkeit der Patientinnen und Patienten wahrgenommen?

C. Abschluss

- Gibt es Aspekte, die Ihnen noch wichtig sind und wir noch nicht thematisiert haben?
- Haben Sie noch Fragen?
