# Supplementary material for: From inpatient to outpatient mental health care: Protocol for a randomised feasibility trial of a care transition intervention for patients with depression and anxiety (the AMBITION-trial)
Source: PLoS One. 2023 Nov 3;18(11):e0291067. doi: 10.1371/journal.pone.0291067 (PMC10624294; doi:10.1371/journal.pone.0291067)
Supplement: S5 File — (PDF) [file pone.0291067.s005.pdf]

Ethikkommission der Med. Fak. HD | Alte Glockengießerei 11/1 | 69115 Heidelberg

Herrn Dr. med. Markus Haun
Universitätsklinikum Heidelberg
Klinik für Allgemeine Innere Medizin
und Psychosomatik
Thibautstrasse 4
69115 Heidelberg

Universität Heidelberg
Ethikkommission der
Med. Fakultät

Alte Glockengießerei 11/1
69115 Heidelberg

Tel. +49 6221 56264-60 (Zentrale)
Fax +49 6221 56264-80
ethikkommission-I@med.uni-heidelberg.de

www.medizinische-fakultaet-hd.uni-heidelberg.de/ethikkommission

Vorsitz:
Prof. Dr. med. Dr. h.c. Thomas Strowitzki

Stellv. Vorsitz:
Prof. Dr. med. Klaus Herfarth

Geschäftsleitung:
Dr. med. Verena Pfeilschifter

eFeedback
Scan or click

04.05.2022
ts-sn

BERUFSRECHTLICHE BERATUNG

Unser Zeichen:	**S-259/2022 (Bitte stets angeben)**
Titel:	**Übergang von stationärer in AMBulante psychosoziale Versorgung – Machbarkeit einer intersektoralen Care Transition IntervenTION bei Depression und Angst (AMBITION)**

Sehr geehrter Herr Dr. Haun,

die Ethikkommission hat Ihr Forschungsvorhaben in der Sitzung am 11.04.2022 beraten. Die zusätzlich angeforderten Informationen sind am 25.04.2022 und am 03.05.2022 eingegangen.

Die Ethikkommission hat keine Bedenken gegen die Durchführung der Studie.

Sie gibt jedoch folgende Empfehlungen bzw. Hinweise:

Allgemein:

1. Der guten Ordnung halber weist die Kommission darauf hin, dass Foto- und Videoaufnahmen allenfalls dann als anonymisiert bzw. pseudonymisiert gelten, wenn nur Ausschnitte (Wunde etc.) oder das Innere des Körpers gezeigt werden. Dabei ist die Zuordenbarkeit der Bilder zur Person in der Regel möglich. Dann liegen keine anonymisierten bzw. pseudonymisierten Bilder vor. Auch der oft verwendete „schwarze Balken" über den Augen, bzw. Pixelung des Gesichts reicht nicht aus. Die Zulässigkeit der Verwendung und Nutzung der Aufnahmen richten sich nach dem Inhalt der Einwilligung des Abgebildeten. Die Studienteilnehmer sollten in der Informationsschrift diesbezüglich aufgeklärt werden.
2. Es wird darauf hingewiesen, dass zukünftige Fragestellungen/Studien auf Grundlage der im Rahmen dieser Studie verwendeten Daten als Neuanträge bei der Ethikkommission einzureichen sind, sofern der aktuelle Antrag diese Aspekte nicht beinhaltet.

Studienprotokoll:

3. Auf S. 25 und S. 26, Abschnitt „Anhang A / B", jeweils unter Punkt A wurde im geänderten Dokument das Wort Video entfernt. Dies widerspricht die Angaben in den restlichen Studiendokumenten. Dieser Widerspruch in Bezug auf Videoaufnahmen sollte behoben werden.

Informationsschrift für Care Transition Navigator:

4. S. 3, Abschnitt "Welche Risiken....": An dieser Stelle sollten die Risiken der Empfänger des Dokumnets (hier CTN) angesprochen werden.

5. S. 3, Abschnitt "Informationen zum Datenschutz:.." Die erste zwei Sätze und der letzte Abschnitt sollten auf die Teilnehmergruppe (CTN) angepasst werden, zumal von der Gruppe keine medizinische Befunde erhoben werden.

Einwilligungserklärung (Version für Care Transition Navigator):

6. Das Dokument sollte für die Teilnehmergruppe angepasst werden: S. 1 und 2: Alle unzutreffende Angaben bzgl. Gesundheitsdaten und Entbindung vom Schweigepflicht sollten entfernt bzw. an die Teilnehmergruppe (hier CTN) angepasst werden.

Wir wünschen Ihnen bei der Durchführung der Studie viel Erfolg.

Bitte leiten Sie das Ergebnis der berufsrechtlichen Beratung und die studienrelevante Korrespondenz allen teilnehmenden Ärzten in unserem Zuständigkeitsbereich weiter.

Mit freundlichen Grüßen

Digital unterschrieben von Dr. Thomas Strowitzki
DN: c=DE, cn=Dr. Thomas Strowitzki, title=Dr., givenName=Thomas Franz, sn=Strowitzki, serialNumber=DTR210096304P0001
Datum: 2022.05.04 12:22:34 +02'00'

Prof. Dr. med. Dr. h.c. Thomas Strowitzki
Vorsitzender

Anlagen
Anhang

©EKHD-ZB-S-DV-070-220401

Allgemeine Hinweise:

- Änderungen in Organisation und Ablauf der Studie sind der Kommission, zusammen mit einer Bewertung der Nutzen-Risiko-Relation, umgehend mitzuteilen. Sowohl die **Antragsnummer** als auch die **geänderten Passagen** sollten in den betreffenden Unterlagen **deutlich gekennzeichnet** sein, da anderenfalls keine zügige Bearbeitung möglich ist.
- Es ist **kontinuierlich und eigenverantwortlich** eine **Nutzen-/Risiken-Abwägung** in Bezug auf die derzeitige **Covid-19-Pandemie** durchzuführen. Dabei ist der Nutzen der Studiendurchführung während der aktuellen Pandemie dem Risiko von Neuinfektionen (der Studienteilnehmer und des Studienpersonals sowie indirekt auch weiterer Personen) gegenüberzustellen. Das Infektionsrisiko z.B. durch studienbedingte Kontakte der Beteiligten oder durch studienbedingten Reiseaufwand ist dabei stets gemäß der aktuellen Pandemiesituation zu bewerten. Sollte die Studie regelmäßige Maßnahmen erfordern (regelmäßige Visiten, regelmäßige Behandlung/Verabreichung von Arzneimitteln o.Ä.), ist auch zu bedenken, ob und ggfs. inwieweit diese Maßnahmen während der Pandemie zuverlässig durchgeführt werden können. Dabei sind auch mögliche Vorkehrungen zur Risikominimierung zu treffen. Sofern aufgrund der aktualisierten Nutzen-/Risiken-Bewertung pandemiebedingt Maßnahmen eingeleitet werden müssen und es dadurch zu Änderungen bei der Studiendurchführung kommt, sind diese Änderungen (insbesondere an Prüfplan und Informationsschrift bzw. Einwilligungserklärung) der Ethikkommission gemäß § 10 Abs. 1 GCP-V als nachträgliche Änderung vorzulegen. Es wird ausdrücklich darauf hingewiesen, dass alle Studienteilnehmer über alle für sie relevanten Änderungen im Studienablauf schriftlich und ohne Verzug informiert werden müssen. § 11 der GCP-V regelt die ggfs. unverzüglich zu ergreifenden Maßnahmen zum Schutz vor unmittelbarer Gefahr. Es empfiehlt sich, alle Abweichungen vom Prüfplan, die auf die Pandemiesituation zurückzuführen sind, zu dokumentieren. Zusätzlich sind immer die Informationen des Arbeitskreises Medizinischer Ethik-Kommissionen in der Bundesrepublik Deutschland e.V. unter www.ak-med-ethik-komm.de zu beachten.
- Jedes Forschungsvorhaben, an dem Versuchspersonen beteiligt sind, ist vor der Rekrutierung der ersten Versuchsperson in einer öffentlich zugänglichen Datenbank zu registrieren.
- Innerhalb von einem Jahr nach Studienende sollte die Studienleitung der Kommission einen Abschlussbericht vorlegen, der eine Zusammenfassung der Ergebnisse und Schlussfolgerungen der Studie enthält, unabhängig davon, ob diese vollständig abgeschlossen oder vorzeitig beendet wurde. Dafür ist die auf der Homepage der Kommission abrufbare Mustervorlage „Abschlussbericht“ zu verwenden (Pfad: -> Sonstige Studien -> Vorlagen).
- Datenschutzrechtliche Aspekte von Forschungsvorhaben werden durch die Ethikkommission grundsätzlich nur kursorisch geprüft. Dieses Votum / diese Bewertung ersetzt mithin nicht die Konsultation des zuständigen Datenschutzbeauftragten. Die Einhaltung der einschlägigen Datenschutzgesetze sowie die Umsetzung des Datenschutzkonzeptes liegen in der Verantwortung des Studienleiters/Prüfers bzw. Sponsors.
- Die Ethikkommission geht davon aus, dass im Falle von Videovisiten die Vorgaben gemäß Anlage 31b Bundesmantelvertrag eingehalten werden und die Visiten mittels eines zertifizierten Anbieters durchgeführt werden.
- Mit dem Urteil des Europäischen Gerichtshofs vom 16. Juli 2020 [Aktenzeichen C-311/18] stellen die Regelungen des EU-US-Privacy Shield insbesondere vor dem Hintergrund des Clarifying Lawful Overseas Use of Data Act (CLOUD Act) bzw. des Foreign Surveillance Act (FISA) keinen geeigneten Rechtsrahmen mehr dar. Es sollte daher seitens der Verantwortlichen im Einzelfall geprüft werden, inwieweit personenbezogene/personenbeziehbare Daten (also auch i.S.d. Art. 4 Abs. 5 DSGVO pseudonymisierte Datensätze) rechtssicher entweder auf Basis geeigneter Garantien (etwa verbindlicher Unternehmensregeln, Standardvertragsklauseln oder auf Basis einer ausdrücklichen Einwilligung nach erfolgter Risiko-Aufklärung nach Art. 49 Abs. 1 lit. a) DSGVO) übermittelt werden können. Es empfiehlt sich v.a. hinsichtlich der Standardvertragsklauseln die Auswirkungen des Urteils und die voraussichtlich folgenden regulatorischen Leitlinien seitens der zuständigen Behörden aufmerksam zu verfolgen.
- Die Ethikkommission der Medizinischen Fakultät Heidelberg arbeitet gemäß den nationalen gesetzlichen Bestimmungen und den ICH-GCP-Richtlinien. Ihren Beratungen liegt die Deklaration des Weltärztebundes von Helsinki in der jeweils aktuellen Fassung zugrunde.
- Unabhängig vom Beratungsergebnis macht die Ethikkommission Sie darauf aufmerksam, dass die ethische und rechtliche Verantwortung für die Durchführung einer Studie beim Leiter der Studie und bei allen teilnehmenden Ärzten liegt.

©EKHD-ZB-S-DV-070-220401

Anhang

Liste der eingereichten Unterlagen

**Primär eingereichte Unterlagen:**	Anschreiben vom 25.03.2022<br>Zusammenfassung<br>Checkliste Sonstige Studien<br>Formular für Erstantrag<br>Informationsschrift für Patientinnen und Patienten Version 1.0 vom 25.03.2022<br>Einwilligungserklärung (Version für Patientinnen und Patienten) Version 1.0 vom 25.03.2022<br>Studienprotokoll Version 1.0 vom 25.03.2022<br>Anhang A. Interviewleitfaden für Interviews mit Patient*innen zu t2<br>Anhang B. Interviewleitfaden für Interviews mit der/dem Care Transition Navigator zu t2<br>Nutzen-/Risiken-Abwägung in Bezug auf die derzeitige COVID-19-Pandemie vom 25.03.2022<br>CV Dr. med. Markus W. Haun, M.Sc. Psych., M.B.A. vom 11.02.2022
**Inhaltliche Nachreichung:**	Nachreichung vom 25.04.2022:<br>Informationsschrift für Patientinnen und Patienten Version 1.1 vom 25.04.2022 (mit und ohne Markierung der Änderungen vs Version 1.0 vom 22.04.2022)<br>Informationsschrift für Care Transition Navigator Version 1.0 vom 25.04.2022<br>Einwilligungserklärung (Version für Care Transition Navigator) Version 1.0 vom 25.04.2022<br>Studienprotokoll Version 1.1 vom 25.04.2022 (mit und ohne Markierung der Änderungen)<br>Inhaltliche Nachforderung S-259/2022 vom 13.04.2022

©EKHD-ZB-S-DV-070-220401
