# Supplementary material for: From inpatient to outpatient mental health care: Protocol for a randomised feasibility trial of a care transition intervention for patients with depression and anxiety (the AMBITION-trial)
Source: PLoS One. 2023 Nov 3;18(11):e0291067. doi: 10.1371/journal.pone.0291067 (PMC10624294; doi:10.1371/journal.pone.0291067)
Supplement: S6 File — (PDF) [file pone.0291067.s006.pdf]

Ethics Committee of the Med. HD | Alte Glockengießerei 11/1 | 69115 Heidelberg

Markus Haun, MD, Heidelberg
University Hospital Department of
General Internal Medicine and
Psychosomatics
Thibautstrasse 4
69115 Heidelberg

University of Heidelberg
Ethics Committee of the
Faculty of Medicine

Alte Glockengiesserei 11/1
69115 Heidelberg

Phone +49 6221 56264-60 (head office)
Fax +49 6221 56264-80
ethikkommission-I@med.uni-heidelberg.de

www.medizinische-fakultaet-hd.uni-heidelberg.de/ethics-commission

Chair:
Prof. Dr. med. Dr. h.c. Thomas Strowitzki

Vice Chair:
Prof. Dr. med. Klaus Herfarth

Management:
Dr. med. Verena Pfeilschifter

eFeedback
Scan or click

04.05.2022
ts-sn

PROFESSIONAL LEGAL ADVICE

Our sign: **S-259/2022 (Please always specify)**
Title: **Transition from Inpatient to AMBulant Mental Health Care - Feasibility of an Intersectoral Care Transition IntervenTION for Depression and Anxiety. (AMBITION.)**

Dear Dr. Haun,

the ethics committee has discussed your research project in the meeting on 11.04.2022. The additional information requested was received on 25.04.2022 and 03.05.2022.

The Ethics Committee has no objections to the conduct of the study.

However, it does provide the following recommendations or guidance:

General:

1. For the sake of good order, the Commission would like to point out that photographs and video recordings are at most considered anonymized or pseudonymized if only sections (wound, etc.) or the inside of the body are shown. In this case, it is generally possible to assign the images to the person. In this case, there are no anonymized or pseudonymized images. Even the often used "black bar" above the eyes, or pixelation of the face is not sufficient. The permissibility of the use and exploitation of the images depends on the content of the consent of the person depicted. The study participants should be informed about this in the information leaflet.
2. It should be noted that future questions/studies based on the data used in this study should be submitted as new applications to the Ethics Committee if the current application does not include these aspects.

Study protocol:

3. On p. 25 and p. 26, section "Appendix A / B", in each case under item A, the word video was removed in the amended document. This contradicts the information in the rest of the study documents. This contradiction regarding video recordings should be corrected.

Care Transition Navigator Informational Guide:

4. P. 3, "What risks. ...." section: At this point, the risks of the recipients of the documnets (here CTN) can be addressed.

5. P. 3, section "Information on data protection:.." The first two sentences and the last paragraph should be adapted to the participant group (CTN), especially since no medical findings are collected from the group.

Consent form (Care Transition Navigator version):

6. The document should be adapted for the participant group: P. 1 and 2: All inaccurate information regarding health data and release from confidentiality should be removed or adapted to the participant group (here CTN).

We wish you every success in carrying out the study.

Please forward the results of the professional consultation and study-related correspondence to all participating physicians in our jurisdiction.

With kind regards

Digitally signed by Dr. Thomas Strowitzki
DN: c=DE, cn=Dr. Thomas Strowitzki, title=Dr., givenName=Thomas Franz, sn=Strowitzki, serialNumber=DTR210096304P0001 Date: 2022.05.04 12:22:34 +02'00'

Prof. Dr. med. Dr. h.c. Thomas Strowitzki
Chairman

Attachments
Appendix

©EKHD-ZB-S-DV-070-220401

General notes:

- Changes in the organization and conduct of the study must be reported to the Commission immediately, together with an assessment of the benefit-risk ratio. Both the **application number** and the **amended passages** should be **clearly marked in** the relevant documents, otherwise no rapid processing is possible.
- A **benefit/risk assessment with regard to** the current **Covid 19 pandemic** must be carried out **continuously and independently. The** benefit of conducting the study during the current pandemic must be weighed against the risk of new infections (of the study participants and study personnel and indirectly also of other persons). The risk of infection
  e.g. through study-related contacts of the participants or through study-related travel expenses must always be evaluated according to the current pandemic situation. If the study requires regular measures (regular visits, regular treatment/administration of drugs, etc.), it must also be considered whether and, if so, to what extent these measures can be reliably implemented during the pandemic. Possible precautions for risk minimization must also be taken. If measures have to be initiated due to the pandemic as a result of the updated benefit/risk assessment, and if this leads to changes in the conduct of the study, these changes (in particular to the study plan and information document or informed consent form) must be submitted to the Ethics Committee as a subsequent amendment in accordance with Section 10, Paragraph 1 GCP-V. It is expressly pointed out that all study participants must be informed in writing and without delay about all changes in the study procedure that are relevant to them. § Section 11 of the GCP-V regulates the measures to be taken immediately, if necessary, to protect against immediate danger. It is recommended to document all deviations from the study plan that are due to the pandemic situation. In addition, the information provided by the Working Group of Medical Ethics Committees in the Federal Republic of Germany (Arbeitskreis Medizinischer Ethik-Kommissionen in der Bundesrepublik Deutschland e.V.) at www.ak-med-ethik-komm.de should always be observed.
- Any research project involving subjects must be registered in a publicly accessible database prior to recruitment of the first subject.
- Within one year after the end of the study, the study management should submit a final report to the Commission containing a summary of the results and conclusions of the study, regardless of whether it was completed in full or terminated prematurely. For this purpose, the sample template "Final Report" available on the Commission's homepage should be used (path: -> Other studies -> Templates).
- Aspects of research projects relating to data protection law are only examined by the ethics committee in a cursory manner. This vote/assessment therefore does not replace consultation with the responsible data protection officer. Compliance with the relevant data protection laws and the implementation of the data protection concept are the responsibility of the study director/investigator or sponsor.
- The Ethics Committee assumes that in the case of video visits, the specifications in accordance with Annex 31b of the Federal Mantel Agreement are complied with and that the visits are carried out by means of a certified provider.
- With the ruling of the European Court of Justice of July 16, 2020 [case number C-311/18], the regulations of the EU-US Privacy Shield no longer represent a suitable legal framework, especially against the background of the Clarifying Lawful Overseas Use of Data Act (CLOUD Act) or the Foreign Surveillance Act (FISA). Therefore, data controllers should examine on a case-by-case basis the extent to which personal data (i.e., also pseudonymized data sets within the meaning of Article 4 (5) of the GDPR) can be transferred in a legally secure manner either on the basis of suitable safeguards (such as binding corporate rules, standard contractual clauses, or on the basis of explicit consent after risk disclosure pursuant to Article 49 (1) (a) of the GDPR). It is advisable to closely monitor the effects of the judgment and the regulatory guidelines likely to follow from the competent authorities, especially with regard to the standard contractual clauses.
- The Ethics Committee of the Heidelberg Medical Faculty operates in accordance with national legal requirements and ICH-GCP guidelines. Its deliberations are based on the Declaration of the World Medical Association of Helsinki as amended from time to time.
- Regardless of the outcome of the consultation, the Ethics Committee draws your attention to the fact that the ethical and legal responsibility for conducting a study lies with the study director and all participating physicians.

©EKHD-ZB-S-DV-070-220401

Appendix

List of submitted documents

**Primary Submitted Documents:**	Cover Letter 03/25/2022<br>Summary Checklist Other<br>Studies Initial Application<br>Form<br>Information leaflet for patients Version 1.0 of 25.03.2022 Informed consent (version for patients) Version 1.0 of 25.03.2022<br>Study protocol version 1.0 from 25.03.2022<br>Appendix A. Interview guide for interviews with patients at t2<br>Appendix B. Interview guide for interviews with the Care Transition Navigator at t2.<br>Benefit/risk trade-off related to the current COVID-19 pandemic of 03/25/2022.<br>CV Markus W. Haun, M.Sc. Psych., M.B.A. dated 11.02.2022
**Content Submission:**	Submission of 04/25/2022:<br>Patient information document version 1.1 dated 04/25/2022 (with and without changes marked vs version 1.0 dated 04/22/2022) Information document for Care Transition Navigator version 1.0 dated 04/25/2022 Informed consent form (version for Care Transition Navigator) version 1.0 dated 04/25/2022<br>Study protocol version 1.1 from 25.04.2022 (with and without marking of changes)<br>Substantive demand S-259/2022 of 13.04.2022

©EKHD-ZB-S-DV-070-220401
