# Supplementary material for: From inpatient to outpatient mental health care: Protocol for a randomised feasibility trial of a care transition intervention for patients with depression and anxiety (the AMBITION-trial)
Source: PLoS One. 2023 Nov 3;18(11):e0291067. doi: 10.1371/journal.pone.0291067 (PMC10624294; doi:10.1371/journal.pone.0291067)
Supplement: Attachment — Submitted filename: renamed_efdb1.docx [file pone.0291067.s007.docx]

# COMMENTS – Manuscript PONE-D-23-11362

Title: “From inpatient to outpatient mental health care: protocol for a randomised feasibility trial of a care transition intervention for patients with depression and anxiety (the AMBITION-trial)”

**Important note: This review pertains only to ‘statistical aspects’ of the study and so ‘clinical aspects’ [like medical importance, relevance of the study, ‘clinical significance and implication(s)’ of the whole study, etc.] are to be evaluated [should be assessed] separately/independently. Further please note that any ‘statistical review’ is generally done under the assumption that (such) study specific methodological [as well as execution] issues are perfectly taken care of by the investigator(s). This review is not an exception to that and so does not cover clinical aspects {however, seldom comments are made only if those issues are intimately / scientifically related & intermingle with ‘statistical aspects’ of the study}. Agreed that ‘statistical methods’ are used as just tools here, however, they are vital part of methodology [and so should be given due importance]. I look at the manuscript in/with statistical view point, other reviewer(s) look(s) at it with different angle so that in totality the review is very comprehensive. However, there should be efforts from authors side to improve (may be by taking clues from reviewer’s comments). Therefore, please do not limit the revision only (with respect) to comments made here.**

**COMMENTS:** Although this manuscript is well drafted [and the study is excellent with respect to most of the aspects (This study being ‘pilot’ in nature, sample size is not a big issue anyway)], I have few observations/concerns (different opinion) which are given below:

Since (as you have mentioned) in ‘Introduction’ section that “Systematic reviews on CTIs show a potential benefit for patients regarding risk of readmissions and symptom severity [14–17]”, and later in ‘Intervention’ section you also state that “optional components have been shown to be effective in several comparable studies [14,17].”, I wonder if there is really a need of such a ‘feasibility trial’? [particularly because the quoted studies are published in reputed journals and after looking at the competence/knowledge of the topic/preparation of the authors]. They could have planned a larger main/final phase-III trial (as said sufficiently powered randomised controlled trial) as well, I guess.

In my knowledge the primary purpose of publishing a research protocol (of main study/trial) is a means to allow the academic community to evaluate whether subsequent analysis and results are in line with the investigators' initial objectives. Additionally, it informs the academic community on ongoing research and may avoid duplication of work. Agreed that making study protocols publicly available has the benefit of disseminating the most contemporary ideas with respect to study design and data analysis. However, the purpose/intention of ‘protocol for a pilot/feasibility study/trial’ is/are not known {at least to me and may be to many others as well}. Will these learned authors may please explain?

[Note that the statement “Patient recruitment will start in July 2022 and will probably be completed in July 2023” in this manuscript prompted me to write the above as this excellent study will get delayed].

Except these minor points, the article is acceptable. However, mind you that as pointed out in ‘important note’ above “This review pertains only to ‘statistical aspects’ of the study and so ‘clinical aspects’ should be assessed separately/independently. ‘Minor Revision’ is recommended.
